# The relationship between income and assets in farms and context of sustainable development

**DOI:** 10.1371/journal.pone.0265128

**Published:** 2022-03-14

**Authors:** Aleksander Grzelak

**Affiliations:** Department of Macroeconomics and Agricultural Economics, Poznań University of Economics and Business, Poznań, Poland; Wroclaw University of Economics and Business Faculty of Economics and Finance: Uniwersytet Ekonomiczny we Wroclawiu Wydzial Ekonomii i Finansow, POLAND

## Abstract

The objective of this paper is to identify the relationship between farm income and assets within the European Union (EU) in the context of economic and environmental sustainable development. The scientific context is connected to economic theory (the recognition of the nature of such a relationship, as well as the determination of whether sustainable development acts as a stimulant or destimulant under these conditions). The Farm Accounting Data Network system was employed in the article. The econometric models were estimated by panel data based on the reported results of the farms operations in EU member states for the period of 2004–2018. Accordingly, the relationship between income and assets is positive and statistically significant, but not very clear in the group of surveyed farms. Moreover, economic sustainability was found to positively influences the relationship between income and assets. In contrast, the relationship between incomes and assets was weakened. The situation is brought about by the intrinsic growth in the value of the land, as well as by the growing importance of non-productive assets. Thus, farmers in EU countries are becoming wealthier in terms of the value of their assets but, this is not reflected directly in their income. The reason is the growing importance of environmental and social functions in the agriculture the European Union. Greater skills in asset management at farm level are, therefore, required to mitigate the situation.

## Introduction

The accumulation of assets is one of the determinants of agricultural development processes. This mainly refers to the process of depositing the income generated in agriculture through investments to maintain the continuity of farm operations. Additionally, increasing the value of assets at the farm level determines economic viability. The relationship between income and assets develops through a sequence of actions: income–savings–propensity to invest–investment–productive and non-productive assets (accumulation). In the article, this approach has been extended via a focus on sustainable development: sustainability (economic and environmental)—income–savings–propensity to invest–investment–productive and non-productive assets (accumulation). The analysed problems also correspond to the broader concept of a circular economy, which emphasises the optimisation of resource management [1], and thus involves the economics of sustainable development [2–4].

In the present situation, possibility exists of increasing the value of farm assets due to the intrinsic growth in the price of agricultural land. However, according to research [5], farmers’ investment behaviour, and thus the relevant asset–income relationship, escapes classical economic theories. If we take into account issues of sustainable development, a question then arises: Is there a conflict between the economic (high income, larger assets) and environmental objectives in agricultural holdings [6]?

The objective of the paper is to identify the relationship between farm assets and income in EU21 countries by considering the context of economic and environmental sustainable development. Currently, the issue of the paradigm of sustainable development in agriculture is becoming increasingly important, both in scientific and practical terms. Contrary to appearances, the relationship is not entirely obvious due to, for example, the possibility of an intrinsic growth in agricultural land prices (not resulting from an increase in the productivity of land). The capitalisation of subsidies [7], and the relatively constant demand for land in a situation of fixed supply are important here.

The research hypotheses were formulated as follows:

**H1:** *The relationship between income and assets (capital) is clear in the group of surveyed farms*.
**H2:** *Economic sustainability positively influences the relationship between farm income and assets (capital)*
**H3**: *Environmental sustainability has a negative impact on the relationship between farm income and assets (capital)*.

In the case of H1, the term `clear`means that variable `income`is statistically significant in the analysed econometric panel models. Simultaneously the relative impact of income on assets (capital), by the prism of the coefficient of regression after standardisation is the highest, as compared to the value of such coefficient for other variables.

The motivation for the study is the existence of a research gap in the relationship between agricultural income and assets that takes the context of economic and environmental sustainability into account. A second motivation is to generate better understanding of current farm development mechanisms. As Hill [8] pointed out, changes in the value of farm assets are often left out while the analyses focus on income issues.

There are also trends in agriculture in the EU relating to increase in agricultural land prices and capitalisation of support in agricultural land prices [9], and the increasing emphasis on the environmental context [10, 11]. The importance of non-productive assets in farms in EU countries is growing as well [12]. So farmers in the EU are becoming wealthier in terms of the value of assets. However, this is not reflected in their income. In sum, references to the discussed problems can be also found in Piketty’s [13] book in terms of links between wealth and profits.

There are both scientific and practical dimensions to the regarded issue. The first refers to the recognition of the nature of such a relationship, as well as to the determination of whether sustainable development is a stimulant or a destimulant in these cases. In contrast, the practical dimension refers to the recommendations for the functioning of farms and the direction of changes due to the EU’s Common Agricultural Policy (CAP) concerning sustaining the economic vitality of farms. The context of the on-going discussion on further changes in the EU’s CAP also cannot be ignored, as well as the structure of the EU’s budget expenditure.

The article consists of five parts. In the Introduction, the motivation to conduct the research, and its hypotheses are presented; in the Literature review, the current state-of-the-art on the raised issues is included; the applied methodology is then explained. Afterwards, the obtained research results are analysed, and the end of the article, conclusions, reflections and the implications for policy adjustments are presented.

## The literature review

### Theoretical background

The accumulation of assets in the classical approach is based on the production function:

Y=f(K,L,Z),whereY=output,K=capital,L=labour,andZ=land

**Fig 1:**
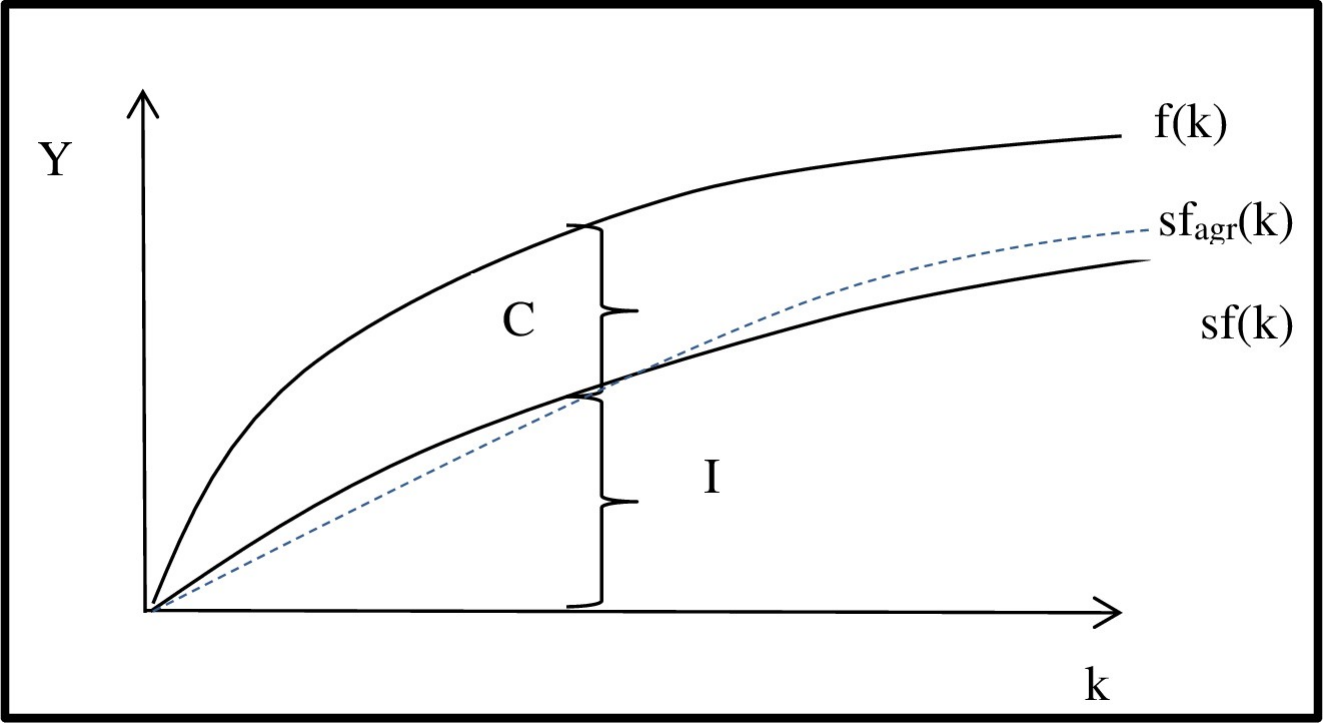
Capital accumulation and growth–The Solow model. Source: based on [14]. S–savings rate, c–consumption, I–investment, k–capital per worker, f_agr_−accumulation function for agriculture.

The transformation of savings that arise from agricultural income into investments undertaken by the agricultural producer refers to Eq 1. In the traditional approach, analogies can be found in the Solow model [14], whereby the greater the amount of capital, the greater the investment (Fig 1). A shortcoming of Solow’s theory is the assumption that other inputs are not relatively important. Consequently, environmental issues are omitted, which are increasingly important at the level of shaping development conditions for farms within the EU (the CAP instruments). There are examples in the literature of the application of Solow’s theory in agriculture [15].

As evidence in Fig 1 the slope of production function is positive but increasingly flattened, this results from the decreasing productivity of capital. Hence, the existence of marginal effects in the context of the relation between profit and capital is relatively universal in economic processes influencing farm operation. However, the uniqueness of the decreasing productivity of capital for farms comes down to the fact that agriculture has a weak market position due to, for example, its distance from the final consumer, as well as generally unfavourable price relationships between the products sold and purchased by farmers [16]. Moreover, there is an increase in the importance of non-productive assets, for example, larger building areas for livestock stands. The increasing role of non-productive assets is a consequence of the need for agricultural producers to meet the requirements for receiving subsidies. As a result, the decreasing productivity of capital acts more restrictively, weakening the relationship between income and assets. In Fig 1, this is reflected by the sf_agr_(k) curve. Its position, above a certain scale of production, is higher than average in the economy due to the intrinsic growth in land prices that increases the value of the assets specially when farmers own their land.

The curve for agriculture (Fig 1) can be considered to be more flattened due to the increased importance of non-productive assets (the need to meet environmental standards), as well as the aforementioned generally unfavourable price relations stated in [16]. In turn, for agriculture, the initial course of the curve results from the fact that for low-scale agricultural outputs, meeting the consumption needs of the farming family takes precedence. Subsequently, regarding investment outlays, the scope of institutionalization of market relations is small and fixed costs are relatively high. Consequently, production effects grow slowly, and the course of the function will change only when a certain threshold of production is exceeded.

In the case of agriculture, we often have to deal with a situation whereby agricultural income alone is sufficient solely to satisfy (not always fully) the consumption needs of the farmer and his family due to the small scale of activity minimal farm income and the domination of non-agricultural sources of income. Thus, there are limited possibilities for financing investments and growth in asset value in agricultural holdings. In consequence, we have the decapitalisation of farm assets, which is additionally aggravated by low creditworthiness or a lack of successors. Analysed issues have been studied in the literature in the context of the so-called `wealth of farm households`[8, 17]. So agricultural activity alone, despite the considerable accumulation of farm assets, is not able to generate income parity. Therefore, the importance of off-farm sources of income increases. Indeed, according to data derived from the Farm Accounting Data Network (FADN) system (EU21), in 2004, the share of non-farm income from farms (agritourism, income from rent, forest, rent of equipment) as agricultural income was 17.1%, while in 2018 it was 24.5%.

Larger farms, which are strongly linked to the market, function on a kind of treadmill. The increase in the value of depreciable assets derived from the desire to improve their competitive position generates ever-greater costs due to the necessity of renewing the assets–higher depreciation costs. In this way the increase in the value of depreciable assets creates pressure for further investments. Part of this type of investment is non-productive or is related to the obligatory fulfilment of requirements under the EU’s CAP, such as the cross-compliance rules in terms of animal welfare. The cross-compliance rules refer i.a. to stock density, For example, [18], based on studies of EU agriculture in the period 1995–2015, found the occurrence of a growth-rate i.e. an increase in real productivity caused a decrease in the growth rate of agricultural income. Hence, while the processes of increase in real productivity stimulate investments, and enhance the value of assets, t they only to a small extent, contribute to the growth of income.

Interesting considerations concerning capital accumulation were presented in [19], where it was pointed out that capital accumulation in agriculture was a function of the past (primary accumulation). Consequently, it is very difficult for smaller farms with a lower level of assets to `catch up`with economically stronger units.

Swinnen and Gow [20] exposed the importance of external sources of financing in capital creation. Thus the value of assets is shaped not only by income, but also by liabilities. In turn, [21] showed that, in the case of farms in the USA, the value of assets in the farm sector between 2009 and 2016 increased by about 34%, while agricultural income only enlarged by about 11%. Similar trends were also reported in earlier studies [22]. In contrast, for farms covered by the FADN system, the median of average annual growth in agricultural income in the EU23 countries was 1.42% over the period 2004–2018, while total assets were 2.49%. Higher growth in the value of assets than in income indicates the functioning of agriculture under conditions of decreasing marginal effects, but also the loosening of the links between assets and income.

### Sustainable development

There are very many proposals for quantification of sustainable development at farm level [23–25], and in the literature we can find theoretical and empirical studies that suggest the existence of synergies between different dimensions of sustainability. For instance, Martínez-Sastrea et al. [26] mention the simultaneous positive effects of animal diversity on pest-control and pollination in apple orchards in Spain.

At present, the operation of agricultural holdings in EU countries is being implemented in conditions that increasingly take into account the context of sustainable development. Therefore, the analysed issues correspond in practical terms currently with a set of initiatives of European Commission to achieve climate neutrality in Europe, the so-called European Green Deal [27]. From the perspective of the relationship, the approach to resource management can be discerned [28, 29]. Among other issues, the idea is to reduce the pressure from farms on the environment while not worsening the productivity of the used resources [30]. At the farm level, not worsening the productivity of resources while reducing the pressure on environment, means that the relationship between income and assets should not be weakened, while environmental sustainability is simultaneously maintained. In practice, however, the objectives of farmers that are linked to economic and environmental dimensions can be contradictory. This can lead to situations in which growing farm income and assets are accompanied by greater pressure on the environment or increasing stratification of incomes and assets among farmers. For example, [31], on investigating sheep farms (in different farming systems) in north-eastern Spain, a clear trade-off between the economic and environmental goals was evident, whereby the higher the economic sustainability, the lower the environmental sustainability. Moreover, [32] underlined how the risk of the negative impact of farms on the environment increased with the growing accumulation and intensification of production. The authors of [33, 34] also affirmed the interchangeability between environmental and economic dimensions in the functioning of farms. In turn [35–37] indicate that a balance between these dimensions is possible and the relationship between economic and environmental objectives is positive.

Different studies stress that larger units, and therefore those with more assets and income, have a better chance of having a positive relationship between the economic and environmental spheres [38]. Moreover, Špička et al. [39], based on the experience of farms in the Czech Republic, underline, that a trade-off between environmental sustainability and economic performance occurs primarily among farming specialisation categories.

### Capitalisation of subsidies

The process of asset accumulation mainly occurs, as mentioned earlier, through income. However, there is another channel through which accumulation occurs – subsidy capitalisation [7, 40]. Direct payments are captured in lease rates and in land. Consequently, the links between income and assets are weaker. The CAP reinforces the pressure on land prices due to decoupled payments. As a result, the rapid growth in land prices has become a significant barrier for farmers who want to increase the scale of their farming activities [41]. For example, as reported by Ciaian et al. [7], in new EU member countries, the capitalisation rate was 79% after the budgetary reform period in 2013. In the old member countries, the capitalisation rate was 43%. The biggest beneficiaries of subsidy capitalisation are landowners, as they have gained from increase value of agricultural land, whereas the actual users i.e. farmers have obstacles because of the increase in lease and land prices.

The importance of the subsidy capitalisation process can be proven by the fact that, after integration with the EU, agricultural land prices in the new member states (e.g. in Poland rose significantly. The data about agricultural land prices published by EUROSTAT indicate that between 2011 and 2019, only in Greece, Lithuania, Slovakia and Italy did average land prices decrease. In contrast, in the remaining countries, there was an increase, and this was significant (more than 1.5-fold) in the Czech Republic, Estonia, Latvia, Luxemburg, Hungary, Poland and Sweden. The additional amenities of land are important here. Moreover, the capitalization of subsidies can create a wealth effect. Farmers may be willing to expand production through activities that they would consider risky in the absence of guaranteed income from direct payments [42]. On the macroeconomic level, therefore, this may stimulate investment in agriculture

## Research methodology

The research methodology used in the article is complex. Therefore it is introduced in Table 1.

**Table 1:** Methodological sequence of the research.

Stages	Explanations
**Selecting a database**	the FADN data https://agridata.ec.europa.eu/extensions/FarmEconomyFocus/FADNDatabase.html
**Selecting dependent variables**	1. the value of total assets (SE436)
	2. the value of capital (SE436- SE446)
**Selecting independent variables**	1. the agricultural income (SE420)
	2. economic sustainability
	3. environmental sustainability
**Selecting control variables**	1. arable land (SE025)
	2. liabilities (long and short term (SE485)
	3. the share of fixed assets to total assets (SE441/SE436)
**Data cleaning**	deflation of variables, regard purchasing power of currency exchange, three-period moving means for variables
**The spatial range of the research**	UE(25)–exclusion of Malta and Cyprus (the marginal importance of their agriculture in the EU)–UE23 (for Fig 2), then also the exclusion of Denmark and Slovakia (high value of assets alongside negative agricultural income in some years in the analysed period)—UE(21) (Tables 2 and 3)
**Selecting appropriate forms of functions**	exponential function
**Selecting method of estimation**	fixed effects (FE) or random effects (RE)

Source: Own compilation based on the referred literature

### Data

In the study, FADN data were employed. The origin of the sources is a website https://agridata.ec.europa.eu/extensions/FarmEconomyFocus/FADNDatabase.html. The mentioned website contains economic and production information about farms covered by the FADN system in the EU Member States. The term `assets`refers, especially in the empirical part, to the value of assets of the economic units (farms) for both current and fixed components. Such definition of `total assets`is in line with the FADN system (fixed assets + current assets) [43]. Only assets in ownership are taken into account, based on the value at closing valuation for the year. As a result, we have the same counting of assets across EU countries, which makes it possible to compare them.

Data of the FADN system are publicly available because of the assured confidentiality of the participating farms and the data are published in the different cross-sections only if there are at least 15 agricultural holdings in the group. Such data are microeconomic and refer to the arithmetic mean of the average farm for a given group of farms (by type of production, economic size, region, country). Thus, the data illustrate the situation of the representative farm for a given country, which is the result of the behaviour of many agricultural producers. The time scope of analysis refers to the period 2004–2018, which was dictated by the availability of data for the studied countries at the time of writing of the article.

The value of total assets was used as a dependent variable (SE436). Total assets, both fixed and current, create and determine the level of income, regardless of whether they are owned by the farmer or not. Moreover, the value of capital in the classical sense (as a factor of production) was used as a dependent variable. In this case, the value of land was subtracted from the value of assets (SE436-SE446). This approach resulted from the intention to possibly identify the impact (in the comparative, indirect sense) of the value of land on the relationship under study. Because there is an intrinsic growth in the value of the land, this complicates the analysis of the relationships between income and assets. This problem is confirmed by many research results [44, 45].

The selection of explanatory variables for the panel models was dictated by substantive considerations. The idea was to include, in addition to the agricultural income variable (SE420) and variables related to the context of economic and environmental sustainability, control variables that have a reasonably consistent theoretical economic impact on the relationships under study [46]. Therefore, the variable of acreage of arable land (SE025), liabilities (long and short term, SE485) and the share of fixed assets to total assets (SE441/SE436) were employed [47, 48].

In the analyses, the context of economic and environmental sustainability was taken into account. The economic sustainability was estimated based on income per 1 hour of work by the farmer’s own family. The level of this income was then compared with the median income of the analysed group of EU countries for a given year. If it was higher than the median, then such an observation was considered to be economically sustainable. Thus, economic sustainability is contractual here. A similar approach can be noted in the case of estimating the so-called sustainable value and setting the benchmark [49].

Environmental sustainability was established based on two sub-measures: the share of cereals in the sowing structure and the intensity of animal stocking per 1 ha of utilised agricultural area (UAA). The choice of these measures was because their use allowed a determination of threshold values that enabled the critical values to be set for the given areas of sustainability. Regarding the share of cereals in the sowing structure, the measure should not exceed 66%. For the stocking density, values in the range of 0.5–1.5 so-called large livestock units [43] per 1 ha UAA are desirable, which is conducive to maintaining appropriate fertiliser management on farms [50, 51]. Hence, these two proposed metrics for environmental sustainability represent both the issues surrounding the diversity of agricultural production and excessive pressure on the environment. This does not mean that they comprehensively address environmental sustainability. However, in the context of the adopted research objectives, this should not be a problem. Furthermore, the applied approach was driven by the availability of data at such an aggregate level.

In the analyses, the original data of the FADN system have been deflated, because indices of changes in income and means of production derived from EUROSTAT data have been applied. In addition, the purchasing power of currency exchange rates was taken into account to eliminate the impact of nominal changes to the exchange rate. At the same time, due to significant variability in economic data of farms, three-period moving averages were used. In the calculation, the mean of the value of the analysed variable for the current year and the previous and next year were employed. For the outlier years i.e. 2004 and 2018, the means respectively, of 2004–2005 and 2017–2018 were applied in order not to lose time series information. In this way the seasonality of data has been reduced.

The explanatory variables in the models were verified by the variance inflation factor (VIF) test for collinearity. Values exceeding 10 indicate the appearance of a collinearity problem. In all models included in the study, the VIF test values did not exceed 5, which means that this problem did not occur in the analysed models [52].

**Fig 2:**
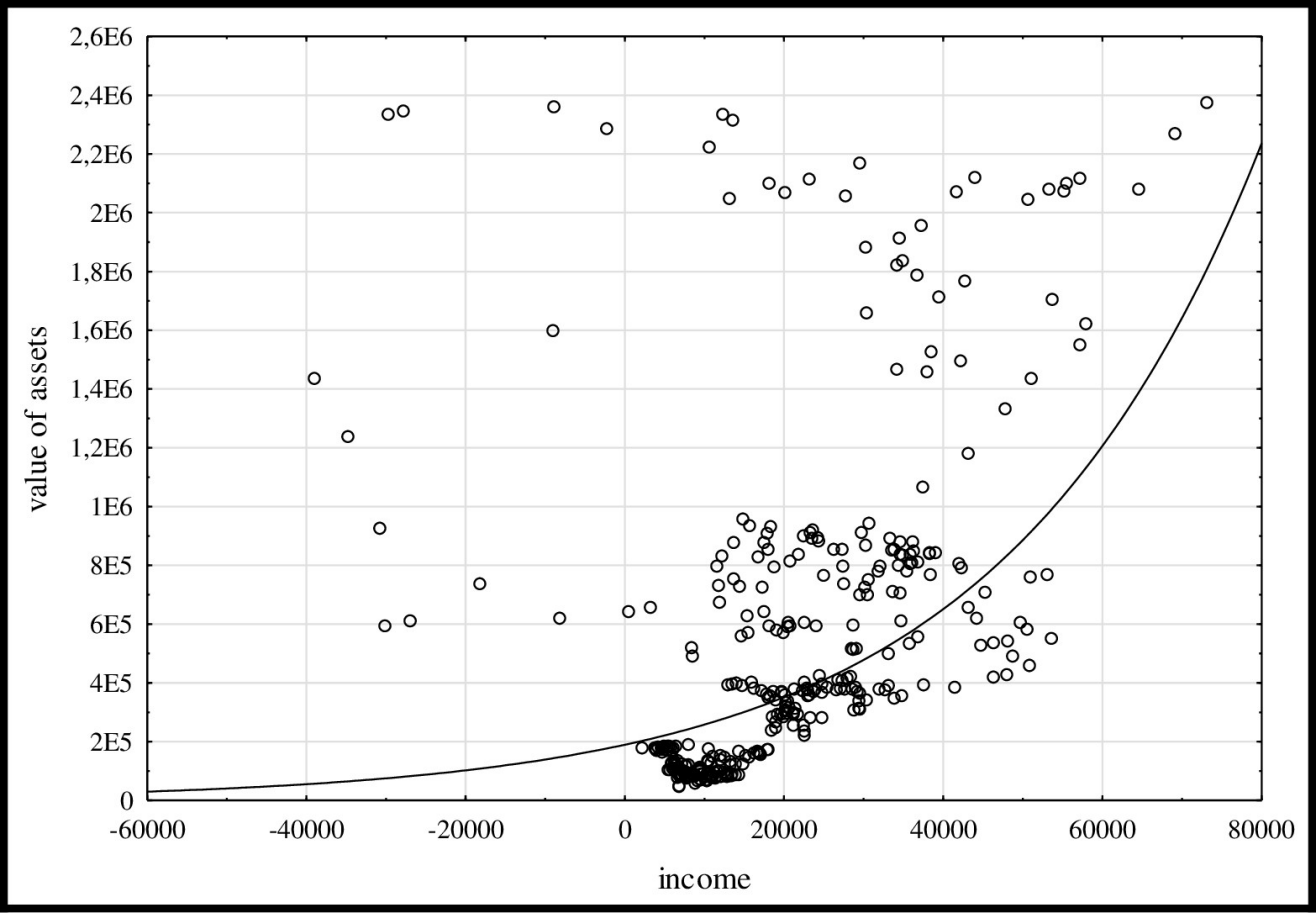
Graphical illustration of the relationship between income (EUR) and assets (EUR; e.g. 1,4E6 means 1,4x10^6^) in farms (FADN system) in the EU countries (23) (2004–2018). Source: own calculation based on data of the FADN system (S1 Table).

The spatial range of the research concerns agricultural holdings from the EU21 countries. From the group of 25 countries, which have been EU members since at least 2004, Cyprus and Malta were excluded due to the marginal importance of their agriculture in the EU. Following preliminary analyses (Fig 2), farms from Denmark and Slovakia were then omitted because those farms recorded high asset values alongside negative agricultural income in the analysed period (2004–2018). Additionally, their farm income showed very high variability. Such effect also distorted the analyses due to the significant levels of achieved values in terms of both income and assets.

### Econometric strategy

The scatter diagram (Fig 2) helped in selecting appropriate forms of functions for models relating to the relationship between income and assets for the examined panel of farms. The analysed observations were concentrated in the lower income and assets value range. Herein, increasing income was accompanied by growing changes in the value of assets. There were, however, quite a few units that were outliers, e.g. farms in Slovakia and Denmark, wherein, especially in some years in the first decade of the 21st century, there were situations where negative income was accompanied by high asset values. From Fig 2 it follows that the most appropriate function for panel regression was an exponential type of function.

The linear functions provided a weak fit to the empirical data, and there was a problem with insignificance with some variables. For the power functions, the fit of the models to the empirical data was similar to the exponential function, but there was statistical insignificance of the variables in some cases. In the case of an exponential function:

$$\hat{Y}=e^{{ß}_{0}+{ß}_{1}x_{1}+{ß}_{2}x_{2}+\cdots +\varepsilon }$$ (1)

after being bilaterally logarithmised, it becomes:

$$ln\;(\hat{Y})=\beta_{0}+\beta_{1}\cdot x_{1}+\beta_{2}\cdot x_{2}+\cdots +\varepsilon$$ (2)

where x_1,_ x_2…_ are explanatory variables, ß_0_ –is the intercept; ß_1_ –structural parameter; ε_it_−random error.

When we have exponential function, then assets change relatively with the income scale. So increasing income has a different effect on the value of farm assets, depending on the scale of income [18, 53]. The exponential function reduces the problems associated with the variance of the random component and the normality of the distribution. Moreover, it allows for more accurate models.

The econometric models were estimated via the panel data, assuming that the development of a dependent variable influences, in addition to the explanatory variables, non-measurable, time-fixed and object-specific factors called `group effects`[54]. In this case, because there are often individual effects, fixed (which we can assign to specific objects) or random panel models with fixed effects (FE) and random effects (RE) are used. The advantage of panel data is that we can control the heterogeneity in the model by considering heterogeneity as constant or random, which solves the endogeneity problem.

To choose an appropriate estimation method, the heteroskedasticity of the random component was assessed. In all analysed models, based on Hausman tests (*p* < 0.05), the null hypothesis of a model with RE was rejected in favour of a model with FE. The rejected model with RE is due to significant across farm differences between the EU countries due to the existence of individual effects. The Wald test was also applied to assess the heteroskedasticity of the random component and the Breusch-Pagan (Br.-Pag.) test [55] was employed to check the constancy of variance of the random component, as well as to verify between classical least squares method estimation and random effects.

In econometric studies the panel model with fixed effects is most often used (also in this paper):

$${\hat{Y}}_{it}=\beta_{0}+\beta_{1}x_{it}+u_{i}+\varepsilon_{it}$$ (3)

where: ß0 –is the intercept; ß_1_ –structural parameter; x–explanatory variable; u_i_−individual effect, ε_it_−random error.

In a panel model with fixed effects, fixed individual effects are eliminated by averaging the model over time (index t) [56]. The Beck–Katz panel-corrected standard error procedure was applied, as this allows us to reduce problems linked to the autocorrelation of the random component.

The use of panel models resulted from the possibility to assess the studied relationships, also taking into account economic, environmental sustainability and control variables. In addition, the use of panel models provides an opportunity to select the form of the function, which was in the article, an exponential function (cf. further consideration).

## Research results and discussion

Descriptive statistics indicate significant differentiation of the studied group in terms of the examined variables (Table 2). All in all, there is a tendency to increase at farm level the value of assets, the capital, agricultural area and, to a lesser extent incomes in the EU countries. As a consequence, we have weakening of the relationship between income and the value of assets.

**Table 2:** Descriptive statistics for the variables used in the panel analyses (2004–2018).

Specification	Mean	Min.	Max.	St. Dev.
**Income** (EUR)	22013	2313	73215	14025
**Total assets** (EUR)	492208	44398	2370979	484339
**Capital (value of total assets–value of land)** (EUR)	229197	24444	757652	175115
**Total utilised agricultural area (SE025)** (ha)	63.6	7.46	231	50.3
**Liabilities** (EUR)	99585	26.67	738071	144461
**Share of fixed assets to total assets** (%)	0.8	0.57	0.95	0.12

Source: own calculation based on data of the FADN system (S2 Table), n = 315

The relationship between assets and income in the group of studied farms turned out to be statistically significant (Table 3). An increase in income by one unit caused an increase in assets by 0.000328%. Furthermore, the variation in assets across countries was much higher than that within the given units (time variable). Hence, the fit of the R2-within model reached a higher value than that of the R2-between model. Higher R2-within is due to the significant heterogeneity of farms and the associated inertial nature of changes in production structures over time.

**Table 3:** The panel models for the relationship between agricultural income and farm assets or capital (FADN system) in EU21 countries (2004–2018; fixed effect estimation). Beck–Katz robust standard errors.

Variable	Model with dependent variable: lntotalassets		Model with dependent variable:lncapital*	
	Coefficient	*p* value	Coefficient	*p* value
**Constant**	11.97	< 0.0001	12.21	< 0.0001
**Income**	3.28e−06 *0*.*05*	0.04	2.85e-06	0.0006
			*0*.*05*	
**Economic sustainability (a)**	0.04	0.02	0.07	0.0008
			*0*.*04*	
	*0*.*02*			
**Environmental sustainability (a)**	−0.04	0.11	−0.04	0.08
			*-0*.*02*	
**Utilised agricultural area**	0.01	< 0.0001	0.01	< 0.0001
	*0*.*51*		*0*.*57*	
**Liabilities**	1.77e−06	< 0.0001	1.3e−06	< 0.0001
	*0*.*26*			
			*0*.*21*	
**Share of fixed assets to total assets**	−0.4	< 0.0001	−1.54	< 0.0001
	*−0*.*04*			
			*−0*.*19*	
Fit assessment and statistical tests				
R2 overall	0.37		0.47	
Within R2	0.57		0.62	
Between R2	0.36		0.46	
Log. of the likelihood	327		324	
Akaike’s inf. criteria	−600		−594	
*p* for Br.-Pag. test	< 0.0001		< 0.0001	
*p* for Hausman test	< 0.0001		< 0.0001	
*p* for Walda test	< 0.0001		< 0.0001	

(a)–com. methodological section; the values of the coefficient after standardisation are written in italics when the variable was statistically significant (< 0.05)

*capital = total assets–value of land

Source: own calculation based on data of the FADN system (S2 Table), n = 315

The regression coefficient for income indicates decreasing scale effects (Table 3). So changes in income values are accompanied by growing changes in assets. Farmers, therefore find it is increasingly difficult in EU countries to generate income effects. Herein, income requires increasing investment outlays, and thus more and more assets, which is linked to decreasing marginal effects. Decreasing marginal effects also result from the run and the exponential function. The point here is that unit changes in income are accompanied by the same percentage changes in assets. Consequently, there is an increasing relationship between changes in assets and changes in income.

In the case of agriculture in EU countries, the faster growth of assets than income is additionally reinforced by increased animal welfare and environmental requirements. Such situation enhanced the importance of non-productive assets. In addition, the intrinsic growth in the price of agricultural land does not result from increasing in productivity with regard to this resource or from inflationary processes [41]. As increase in the value of assets in agricultural holdings results from intrinsic growth in the value of the land, an increase in the importance of non-productive assets or new land utilities is not conducive to an improvement in the income situation. However, the enhanced value of assets by intrinsic growth makes farmers’ creditworthiness increase in terms of securing credits with agricultural land.

In light of previous considerations, a question may arise about the direction of influence of the two sustainability dimensions (economic and environmental) on the relationship between income and assets. The research shows that they have different influences on the value of assets in the group of studied farms. While economic sustainability favours the increase of assets, for environmental sustainability, from the perspective of the share of cereals in the sowing structure and the intensity of animal stocking, the effect is negative. Stronger pro-environmental incentives, including the conditionality of direct payments under the CAP instruments, will reinforce this impact in the future, and at the same time, it will weaken the relationship between income and assets.

The challenge for science here is to identify the impact of pro-environmental incentives on the environmental sustainability of farms and the possible competitiveness of food production in the EU countries compared to other countries. For example, strong asset value responses were recorded for the acreage of the UAA and the value of liabilities in the surveyed farms. Still, for the variable share of fixed assets to total assets, the effect was negative and slightly weaker than for income.

In the case of liabilities, the point is that indebtedness in agriculture is not high compared to other sectors, and the dominant sources of asset creation are agricultural income, subsidies, as well as growth in land value. The data in the Report of the European Commission [57] reveal that, in 2015, did the debt rate (debt/asset value) exceeded 35% only in the agriculture practised in Denmark, France and Slovakia. In contrast, for 11 member countries, the rate did not surpass an average of 10%, which is a very low value.

It should be noted that, as indicated by Barry et al. [58], farmers prefer their own resources in the hierarchy of sources for financing their agricultural activity. In such situation, a small increase in indebtedness may impact relatively high on assets (the importance of the base effect). At the same time, there is a risk that under conditions of increased debt, and farmers’ use of agricultural land as collateral for debt, a wealth effect may arise. Agriculture in the USA, for example, had experienced increasing debt in the 1980`s, resulting in bankruptcies [59]. In contrast, the very high importance of the UAA on the formation of assets is related to direct payments, which are a function of the UAA. Moreover, such a large impact from the arable area results from the fact that part of the arable area (that owned by the farmer) is included in the asset value.

A negative effect of the share of fixed assets to total assets on the value of assets was also noted. The increase of fixed assets limit flexibility of production processes to market conditions. Land is still a basic production factor in agriculture, and its non-productive utilities are, to a greater extent, valorised by the EU CAP instruments more than they were previously. Non-productive amenities influence the heightened value of land. In turn, current assets, directly generated production effects are important elements of the economic basis of farm functioning. They allow for a direct translation into the volume of production and income, and then, through investments, into the accumulation of assets. As shown by the research Skarżyńska et al. [60] conducted on farms in Poland and Lithuania, a higher share of fixed assets in these countries resulted in much lower capital efficiency (more than two times).

Analysis related to capital as dependent variable allow evaluating the assessed relations, excluding the influence of the intrinsic growth increase of the value of assets resulting from changes in land prices. We can observe a more accurate fit to empirical data in model with capital as dependent variable (Table 3). Hence, in such model we have a more clear relationship with the examined dependencies. The increase in the value of assets is also associated with rising land prices (issue of capitalization of subsidies, speculative effect), which weakens the relationship between income and assets. Therefore, excluding land values improved the fit of the models.

The presented processes have their origin in the support instruments of the CAP. There are two issues that are involved: firstly, the capitalisation of subsidies in the price of agricultural land; secondly, such an asset is a safeguard for the implementation of the environmental and social functions of agriculture. The latter was already initiated in the CAP reform of R. MacSharry in the 1990s and was strengthened in the next reforms [61]. To an increasing extent, assets on farms are non-productive due to the need to meet certain environmental welfare standards, which is a condition for receiving payments under the Common Agricultural Policy. Thus, assets generate income to a lesser extent. On the other hand, farms can create public goods and thus also influence the social dimension of agricultural activity.

The research allowed for verification of H1. Thus, the relationship between income and assets is positive, statistically significant, but not very clear compared to the relative impact of other variables such as utilised agricultural area or liabilities in the surveyed group of agricultural holdings. The researched farms operate under conditions of decreasing marginal effects, which means that more and more assets are needed to generate unit income. The assets of agricultural holdings increasingly have a non-productive character relating to the provision of higher requirements for the welfare of the environment, as well as animals, safety, and work comfort. The relationship between income and assets is weakened by the intrinsic growth in the value of the land, which leads to an increase in asset accumulation. The reasons for this process are capitalisation of the subsidies in the heightened value of land and the valorisation of additional land amenities. Furthermore, farmers have greater difficulties in generating income, while there is an intrinsic growth accumulation of assets which does not directly increase their income. In the case of H2, positive confirmation has been made. Economic sustainability has favoured the relationship between income and assets in the group of studied farms. Still, in the case of environmental sustainability (H3), the impact was indeed negative, but this is not of clear statistical significance. So the impact of sustainability on the studied relationship is contradictory, therefore complex, and requires further research. This work also indicates that out of all the control variables, acreage, liabilities and decoupled subsidies had a positive effect on the relationship between income and assets. In contrast, the share of fixed assets to total assets had a negative effect due to the low flexibility of the farms’ production apparatus.

The conducted analyses may suggest that possible contradictions between economic and environmental goals should be mitigated through the CAP instruments. Two possible solutions are to offer green investment grants co-financed from the EU budget or support for the development of alternative energy sources.

The examined relationships will be loosened in the future, which will create challenges for agricultural producers. Among these is the wealth effects associated with agricultural producers’ beliefs about the rise in asset value being due to the intrinsic growth in land value. Such a wealth effect generates increasing speculation in agriculture, especially in the new Member States (Single Area Payment Scheme—SAPS of direct payments place there). With a view to limit the capitalisation of subsidies in the price of agricultural land, a system of direct payments more closely linked to environmental objectives should be considered, as well as a greater degressivity of direct payments in the CAP.

## Conclusions

Referring to the aim of the article, the relationship between farm income and assets in the EU21 countries was found to be statistically significant, but not very clear. Furthermore, this was positively influenced by economic sustainable development. In the light of the presented research results, we can conclude that farmers become wealthier in terms of value of assets, on the other hand, this is not reflected directly in their income. Therefore, non-agricultural rural development is also important because of its potential to diversify the farm family’s income and thus reduce the economic effects of the declining marginal effects of farm operations for income. Thus, the relationship between income and assets, at least at the farm level, is more complex than it might seem in light of Piketty’s [13] results, exposing the issue of assets. Increasing asset value does not yet guarantee increase of income which is related to the growing importance of environmental and social functions in the agriculture of the European Union countries. Non-productive assets are, therefore, increasingly important, and in addition there is a capitalisation of subsidies in the price of agricultural land. Both non-productive assets and capitalization of subsidies require greater qualifications and knowledge in asset management at farm level. Assuming that farmers are rational in their decisions under existing constraints, e.g. informational, the identified relationship between income and assets, reflect a rational choice given the circumstances.

Whether there is a conflict between the economic (high income, larger assets) and environmental objectives in agricultural holdings in terms of the impact of economic and environmental sustainability on the studied relationship, is not clearly resolved. The implication is that farm management is becoming increasingly complex. Due to existing decreasing marginal effects, farmers find it more and more difficult to generate parity income. The mentioned complexity results mainly from policies, also changing of external conditions and has a direct influence on managing a farm. The complexity of the decision process may lead to non-optimal decisions and hurt some farmers (with less education, experience or resources). So improving the education of farmers reduces these problems.

Higher farm`s asset values limit entry for new farmers. Therefore, these problems indirectly relate to inheritance, the generational replacement of farmers and social policy. It remains an open question as to whether the possible rising asset costs will be able to be compensated for by a higher income, also as a consequence of improvement of asset management or possible environmental welfare compensation from the EU CAP instruments.

Further research in the analysed areas should refer to microeconomic analyses at the level of regions with different levels of development. The social dimension of sustainability, including consideration of human capital and income distribution, is interesting. A challenge is to determine the impact of climate change on the examined relationship [62]. Climate change will be associated with an even higher asset intensity of income (the need to invest in sprinklers, the costs of adapting livestock buildings).

An important research limitation is the lack of extended information in the FADN system concerning the non-agricultural income of farmers’ households, as well as the environmental impact of farms. Assessments will then be even more comprehensive and detailed in terms of the analysed problems. Nevertheless, even with the existing possibilities of access to the FADN system data, the research can be successfully continued in the future, especially taking into account regional variations. At the macro level, it will be valuable in the future to relate the analysed relationships more closely to the concept of a circular economy. The point here is to optimise the management of resources (including assets), or to minimise environmental inputs and greenhouse gas emissions.

## Supporting information

S1 Table Income and assets in farms (FADN system) in the EU countries (23). Source: Own calculation based on data of the FADN system.
(PDF)

S2 Table Variables used to the panel models. Source: Own calculation based on data of the FADN system.
(PDF)
