# Supplementary material for: The relationship between income and assets in farms and context of sustainable development
Source: PLoS One. 2022 Mar 14;17(3):e0265128. doi: 10.1371/journal.pone.0265128 (PMC8920205; doi:10.1371/journal.pone.0265128)
Supplement: S1 Table — Source: Own calculation based on data of the FADN system. (PDF) [file pone.0265128.s001.pdf]

S1 Table. Income and assets in farms (FADN system) in the EU countries (23).

Year	Country	Farm Net Income (SE420)	income-ind	inco-ind-equalised
2018	(BEL) Belgium	71029,00	56579,58	53154,79
2018	(CZE) Czech Republi	43439,00	29267,52	29246,34
2018	(DAN) Denmark	5162,00	4302,56	23320,26
2018	(DEU) Germany	38436,00	32323,02	36900,90
2018	(ELL) Greece	9772,00	8530,89	9511,27
2018	(ESP) Spain	34995,00	29470,38	29534,09
2018	(EST) Estonia	8499,00	4651,42	8125,15
2018	(FRA) France	39359,00	33310,01	32069,69
2018	(HUN) Hungary	22132,00	18417,29	18160,60
2018	(IRE) Ireland	24839,00	21682,77	24501,05
2018	(ITA) Italy	37009,00	30224,04	28475,38
2018	(LTU) Lithuania	9514,00	5860,87	7291,06
2018	(LUX) Luxembourg	58394,00	40088,63	39178,70
2018	(LVA) Latvia	9776,00	5607,46	7419,44
2018	(NED) Netherlands	82296,00	69624,87	73214,75
2018	(OST) Austria	32339,00	25041,24	24580,94
2018	(POL) Poland	8943,00	6415,90	6690,59
2018	(POR) Portugal	18584,00	14963,23	14405,39
2018	(SUO) Finland	21599,00	17094,52	15989,32
2018	(SVE) Sweden	9229,00	7273,34	14956,49
2018	(SVK) Slovakia	70290,00	45390,93	25042,86
2018	(SVN) Slovenia	10113,00	8069,27	6549,56
2018	(UKI) United Kingdo	42474,00	32294,54	39619,28
2017	(BEL) Belgium	61523,00	49730,00	51046,06
2017	(CZE) Czech Republi	41845,00	29225,16	28607,51
2017	(DAN) Denmark	50197,00	42337,95	18249,62
2017	(DEU) Germany	48763,00	41478,79	36193,34
2017	(ELL) Greece	11909,00	10491,65	9517,55
2017	(ESP) Spain	34750,00	29597,81	29677,29
2017	(EST) Estonia	20508,00	11598,89	5047,68
2017	(FRA) France	36028,00	30829,38	28726,78
2017	(HUN) Hungary	22055,00	17903,91	18009,54
2017	(IRE) Ireland	31012,00	27319,34	23578,72
2017	(ITA) Italy	32298,00	26726,72	28063,77
2017	(LTU) Lithuania	13769,00	8721,26	7183,60
2017	(LUX) Luxembourg	54495,00	38268,78	35054,03
2017	(LVA) Latvia	15789,00	9231,41	7708,10
2017	(NED) Netherlands	89784,00	76804,63	69225,39
2017	(OST) Austria	30648,00	24120,64	22647,57
2017	(POL) Poland	9629,00	6965,28	6402,38
2017	(POR) Portugal	16959,00	13847,55	13964,04
2017	(SUO) Finland	18526,00	14884,12	14116,50
2017	(SVE) Sweden	26787,00	22639,64	15862,35
2017	(SVK) Slovakia	7196,00	4694,79	35520,11
2017	(SVN) Slovenia	6213,00	5029,84	5685,96
2017	(UKI) United Kingdo	46798,00	46944,01	36846,27
2016	(BEL) Belgium	56954,00	46828,60	45336,07
2016	(CZE) Czech Republi	37162,00	27329,85	28744,00

2016	(DAN) Denmark	9480,00	8108,35	20259,08
2016	(DEU) Germany	40462,00	34778,22	34520,74
2016	(ELL) Greece	10754,00	9530,11	9706,19
2016	(ESP) Spain	34702,00	29963,69	28854,41
2016	(EST) Estonia	-1889,00	-1107,27	4821,50
2016	(FRA) France	25640,00	22040,95	27727,95
2016	(HUN) Hungary	20886,00	17707,40	16795,51
2016	(IRE) Ireland	24396,00	21734,05	24348,64
2016	(ITA) Italy	32695,00	27240,56	26941,70
2016	(LTU) Lithuania	10553,00	6968,69	8031,62
2016	(LUX) Luxembourg	37526,00	26804,69	34667,48
2016	(LVA) Latvia	13761,00	8285,44	8557,21
2016	(NED) Netherlands	70703,00	61246,67	64758,98
2016	(OST) Austria	23595,00	18780,83	19769,96
2016	(POL) Poland	7726,00	5825,96	6151,09
2016	(POR) Portugal	15782,00	13081,33	13544,14
2016	(SUO) Finland	12813,00	10370,85	13067,06
2016	(SVE) Sweden	20832,00	17674,06	18444,64
2016	(SVK) Slovakia	85528,00	56474,62	27485,40
2016	(SVN) Slovenia	4814,00	3958,76	4273,55
2016	(UKI) United Kingdo	32105,00	31300,26	34578,53
2015	(BEL) Belgium	47156,00	39449,61	43249,99
2015	(CZE) Czech Republi	39847,00	29676,99	33286,88
2015	(DAN) Denmark	12026,00	10330,93	13314,51
2015	(DEU) Germany	31397,00	27305,22	32184,18
2015	(ELL) Greece	10290,00	9096,81	9618,59
2015	(ESP) Spain	31171,00	27001,73	24883,33
2015	(EST) Estonia	6653,00	3972,88	2313,04
2015	(FRA) France	35079,00	30313,53	27187,44
2015	(HUN) Hungary	17346,00	14775,23	17006,22
2015	(IRE) Ireland	27012,00	23992,53	23739,95
2015	(ITA) Italy	31875,00	26857,82	27430,25
2015	(LTU) Lithuania	12526,00	8404,93	7348,91
2015	(LUX) Luxembourg	54080,00	38928,97	33649,92
2015	(LVA) Latvia	13428,00	8154,79	7517,36
2015	(NED) Netherlands	64613,00	56225,63	55276,05
2015	(OST) Austria	20276,00	16408,40	17953,31
2015	(POL) Poland	7808,00	5662,01	5950,01
2015	(POR) Portugal	16253,00	13703,53	12930,32
2015	(SUO) Finland	17198,00	13946,21	13579,36
2015	(SVE) Sweden	17653,00	15020,23	13808,97
2015	(SVK) Slovakia	32402,00	21286,80	32001,55
2015	(SVN) Slovenia	4625,00	3832,05	3864,24
2015	(UKI) United Kingdo	27421,00	25491,33	30392,02
2014	(BEL) Belgium	51276,00	43471,76	44340,52
2014	(CZE) Czech Republi	56459,00	42853,81	35914,59
2014	(DAN) Denmark	24885,00	21504,24	27820,11
2014	(DEU) Germany	38968,00	34469,10	35961,76
2014	(ELL) Greece	11611,00	10228,85	9395,83
2014	(ESP) Spain	20305,00	17684,56	21340,61

2014	(EST) Estonia	6746,00	4073,49	6222,51
2014	(FRA) France	33420,00	29207,84	29092,71
2014	(HUN) Hungary	21322,00	18536,02	16385,62
2014	(IRE) Ireland	26627,00	25493,27	23396,88
2014	(ITA) Italy	33149,00	28192,36	24901,83
2014	(LTU) Lithuania	9936,00	6673,10	7941,24
2014	(LUX) Luxembourg	48833,00	35216,10	36255,38
2014	(LVA) Latvia	10064,00	6111,84	6743,84
2014	(NED) Netherlands	55141,00	48355,84	55632,43
2014	(OST) Austria	22552,00	18670,69	18756,12
2014	(POL) Poland	8706,00	6362,06	6435,03
2014	(POR) Portugal	13957,00	12006,10	12560,94
2014	(SUO) Finland	19919,00	16421,03	14858,16
2014	(SVE) Sweden	10327,00	8732,62	12361,55
2014	(SVK) Slovakia	27824,00	18243,24	11912,36
2014	(SVN) Slovenia	4543,00	3801,92	3931,81
2014	(UKI) United Kingdo	36772,00	34384,47	35024,46
2013	(BEL) Belgium	58513,00	50100,18	49800,09
2013	(CZE) Czech Republi	47882,00	35212,96	36874,84
2013	(DAN) Denmark	59108,00	51625,17	41839,34
2013	(DEU) Germany	51177,00	46110,97	42060,75
2013	(ELL) Greece	10247,00	8861,83	9553,06
2013	(ESP) Spain	22249,00	19335,54	18563,48
2013	(EST) Estonia	17086,00	10621,14	10683,58
2013	(FRA) France	31578,00	27756,76	33275,35
2013	(HUN) Hungary	18293,00	15845,62	17065,75
2013	(IRE) Ireland	21650,00	20704,85	22519,41
2013	(ITA) Italy	22900,00	19655,31	23011,91
2013	(LTU) Lithuania	12903,00	8745,68	8820,32
2013	(LUX) Luxembourg	46730,00	34621,07	33431,36
2013	(LVA) Latvia	9650,00	5964,88	6847,47
2013	(NED) Netherlands	70878,00	62315,82	57312,71
2013	(OST) Austria	25048,00	21189,26	21430,15
2013	(POL) Poland	9867,00	7281,02	7217,20
2013	(POR) Portugal	13822,00	11973,18	11992,64
2013	(SUO) Finland	16956,00	14207,23	16378,61
2013	(SVE) Sweden	16296,00	13331,79	11759,18
2013	(SVK) Slovakia	-5796,00	-3792,95	3334,01
2013	(SVN) Slovenia	4950,00	4161,45	4081,13
2013	(UKI) United Kingdo	43901,00	45197,58	42832,07
2012	(BEL) Belgium	64381,00	55828,33	50654,68
2012	(CZE) Czech Republi	45210,00	32557,74	34790,57
2012	(DAN) Denmark	59563,00	52388,62	44093,63
2012	(DEU) Germany	49635,00	45602,19	42397,73
2012	(ELL) Greece	11330,00	9568,50	9572,84
2012	(ESP) Spain	21399,00	18670,35	19043,70
2012	(EST) Estonia	26835,00	17356,09	14452,62
2012	(FRA) France	48386,00	42861,45	37643,29
2012	(HUN) Hungary	19348,00	16815,62	17142,16
2012	(IRE) Ireland	22064,00	21360,12	21926,79

2012	(ITA) Italy	24406,00	21188,07	20290,88
2012	(LTU) Lithuania	16075,00	11042,17	10288,60
2012	(LUX) Luxembourg	40427,00	30456,91	34711,54
2012	(LVA) Latvia	13475,00	8465,68	7625,82
2012	(NED) Netherlands	68802,00	61266,47	53410,41
2012	(OST) Austria	28419,00	24430,52	23990,70
2012	(POL) Poland	10873,00	8008,52	7792,96
2012	(POR) Portugal	13546,00	11998,63	11679,37
2012	(SUO) Finland	21537,00	18507,55	17310,47
2012	(SVE) Sweden	15899,00	13213,14	13765,89
2012	(SVK) Slovakia	-6763,00	-4448,25	632,18
2012	(SVN) Slovenia	5011,00	4280,04	4821,18
2012	(UKI) United Kingdo	48564,00	48914,15	53825,83
2011	(BEL) Belgium	52064,00	46035,52	53673,44
2011	(CZE) Czech Republi	51112,00	36601,02	28794,24
2011	(DAN) Denmark	31364,00	28267,10	29675,13
2011	(DEU) Germany	38051,00	35480,04	38521,13
2011	(ELL) Greece	12228,00	10288,20	10534,42
2011	(ESP) Spain	21946,00	19125,22	19041,92
2011	(EST) Estonia	22860,00	15380,62	15259,87
2011	(FRA) France	47218,00	42311,67	41586,68
2011	(HUN) Hungary	21671,00	18765,23	15694,85
2011	(IRE) Ireland	23950,00	23715,41	20827,17
2011	(ITA) Italy	22719,00	20029,27	20597,21
2011	(LTU) Lithuania	15692,00	11077,95	11192,26
2011	(LUX) Luxembourg	50548,00	39056,65	30374,47
2011	(LVA) Latvia	12793,00	8446,88	8091,31
2011	(NED) Netherlands	40570,00	36648,95	50744,14
2011	(OST) Austria	30037,00	26352,34	23872,37
2011	(POL) Poland	10887,00	8089,35	7838,21
2011	(POR) Portugal	12543,00	11066,30	11419,33
2011	(SUO) Finland	21717,00	19216,61	19860,26
2011	(SVE) Sweden	16951,00	14752,74	14523,38
2011	(SVK) Slovakia	15220,00	10137,76	-7993,58
2011	(SVN) Slovenia	7017,00	6022,04	5451,55
2011	(UKI) United Kingdo	61823,00	67365,76	58056,24
2010	(BEL) Belgium	65713,00	59156,47	48230,45
2010	(CZE) Czech Republi	23382,00	17223,95	20873,24
2010	(DAN) Denmark	9232,00	8369,68	-2086,86
2010	(DEU) Germany	36587,00	34481,15	30665,97
2010	(ELL) Greece	13851,00	11746,55	11072,79
2010	(ESP) Spain	22186,00	19330,19	18703,50
2010	(EST) Estonia	18395,00	13042,88	12148,30
2010	(FRA) France	43761,00	39586,94	32859,71
2010	(HUN) Hungary	13188,00	11503,69	12174,05
2010	(IRE) Ireland	17319,00	17405,99	18916,07
2010	(ITA) Italy	22969,00	20574,30	20393,03
2010	(LTU) Lithuania	15401,00	11456,65	10828,02
2010	(LUX) Luxembourg	26693,00	21609,85	27433,23
2010	(LVA) Latvia	10478,00	7361,36	7056,05

2010	(NED) Netherlands	60014,00	54317,00	37333,39
2010	(OST) Austria	23320,00	20834,26	22458,56
2010	(POL) Poland	9985,00	7416,77	6929,12
2010	(POR) Portugal	12721,00	11193,04	10766,14
2010	(SUO) Finland	24068,00	21856,63	18318,09
2010	(SVE) Sweden	16770,00	15604,27	11986,49
2010	(SVK) Slovakia	-44158,00	-29670,24	-26847,31
2010	(SVN) Slovenia	6980,00	6052,56	6062,79
2010	(UKI) United Kingdo	52641,00	57888,80	57304,47
2009	(BEL) Belgium	43062,00	39499,37	46444,13
2009	(CZE) Czech Republi	11607,00	8794,75	15648,62
2009	(DAN) Denmark	-47048,00	-42897,36	-27633,98
2009	(DEU) Germany	23233,00	22036,71	27620,09
2009	(ELL) Greece	13098,00	11183,60	11614,01
2009	(ESP) Spain	20233,00	17655,09	19749,78
2009	(EST) Estonia	11110,00	8021,40	11128,85
2009	(FRA) France	18244,00	16680,52	29330,45
2009	(HUN) Hungary	6878,00	6253,23	10581,96
2009	(IRE) Ireland	16053,00	15626,82	16829,32
2009	(ITA) Italy	22870,00	20575,52	20332,36
2009	(LTU) Lithuania	13078,00	9949,45	10926,79
2009	(LUX) Luxembourg	25790,00	21633,20	26374,03
2009	(LVA) Latvia	7660,00	5359,91	6759,78
2009	(NED) Netherlands	23023,00	21034,22	34343,29
2009	(OST) Austria	22402,00	20189,07	23079,08
2009	(POL) Poland	6445,00	5281,23	6121,55
2009	(POR) Portugal	11337,00	10039,09	10462,66
2009	(SUO) Finland	15236,00	13881,04	18102,38
2009	(SVE) Sweden	5362,00	5602,44	15517,49
2009	(SVK) Slovakia	-90365,00	-61009,45	-30013,26
2009	(SVN) Slovenia	7124,00	6113,77	5713,02
2009	(UKI) United Kingdo	40424,00	46658,83	51177,12
2008	(BEL) Belgium	44107,00	40676,55	44819,33
2008	(CZE) Czech Republi	28426,00	20927,14	20028,18
2008	(DAN) Denmark	-52705,00	-48374,25	-29613,80
2008	(DEU) Germany	27271,00	26342,39	30269,96
2008	(ELL) Greece	13601,00	11911,87	12184,27
2008	(ESP) Spain	25479,00	22264,07	21824,26
2008	(EST) Estonia	17099,00	12322,27	12649,00
2008	(FRA) France	34676,00	31723,88	29668,36
2008	(HUN) Hungary	16444,00	13988,95	10609,49
2008	(IRE) Ireland	18788,00	17455,16	17636,47
2008	(ITA) Italy	21695,00	19847,25	21264,24
2008	(LTU) Lithuania	15459,00	11374,26	11434,00
2008	(LUX) Luxembourg	42186,00	35879,02	33979,71
2008	(LVA) Latvia	12137,00	7558,08	7848,97
2008	(NED) Netherlands	30230,00	27678,66	30481,74
2008	(OST) Austria	30723,00	28213,90	25499,90
2008	(POL) Poland	8197,00	5666,65	6220,92
2008	(POR) Portugal	11345,00	10155,85	9863,02

2008	(SUO) Finland	20026,00	18569,48	19093,75
2008	(SVE) Sweden	26163,00	25345,76	18238,71
2008	(SVK) Slovakia	831,00	639,90	-18071,98
2008	(SVN) Slovenia	5604,00	4972,71	5919,15
2008	(UKI) United Kingdo	45090,00	48983,74	47892,05
2007	(BEL) Belgium	57763,00	54282,07	48870,63
2007	(CZE) Czech Republi	36342,00	30362,64	24098,51
2007	(DAN) Denmark	2611,00	2430,22	-8778,71
2007	(DEU) Germany	43529,00	42430,77	33730,18
2007	(ELL) Greece	14726,00	13457,32	12887,69
2007	(ESP) Spain	28601,00	25553,62	23445,75
2007	(EST) Estonia	22847,00	17603,34	13387,39
2007	(FRA) France	43354,00	40600,67	34888,10
2007	(HUN) Hungary	13018,00	11586,29	10946,57
2007	(IRE) Ireland	21411,00	19827,44	18038,73
2007	(ITA) Italy	24948,00	23369,94	21275,92
2007	(LTU) Lithuania	16078,00	12978,30	10456,57
2007	(LUX) Luxembourg	50284,00	44426,90	38436,45
2007	(LVA) Latvia	15278,00	10628,92	9594,18
2007	(NED) Netherlands	45620,00	42732,35	38657,92
2007	(OST) Austria	30007,00	28096,75	26701,81
2007	(POL) Poland	9979,00	7714,87	6509,59
2007	(POR) Portugal	10315,00	9394,12	9643,51
2007	(SUO) Finland	25990,00	24830,74	20359,45
2007	(SVE) Sweden	24703,00	23767,92	19184,61
2007	(SVK) Slovakia	7769,00	6153,59	-30652,57
2007	(SVN) Slovenia	7197,00	6670,97	4914,16
2007	(UKI) United Kingdo	50562,00	48033,58	43235,88
2006	(BEL) Belgium	53925,00	51653,26	50941,84
2006	(CZE) Czech Republi	23860,00	21005,75	22705,06
2006	(DAN) Denmark	20543,00	19607,89	12402,74
2006	(DEU) Germany	32681,00	32417,39	34752,62
2006	(ELL) Greece	14065,00	13293,87	13506,60
2006	(ESP) Spain	24374,00	22519,57	22597,14
2006	(EST) Estonia	11819,00	10236,55	14008,98
2006	(FRA) France	33672,00	32339,76	33966,80
2006	(HUN) Hungary	7362,00	7264,46	8145,63
2006	(IRE) Ireland	17960,00	16833,59	18116,67
2006	(ITA) Italy	21470,00	20610,56	21489,37
2006	(LTU) Lithuania	8007,00	7017,16	8912,50
2006	(LUX) Luxembourg	39035,00	35003,42	38443,77
2006	(LVA) Latvia	12678,00	10595,53	10273,52
2006	(NED) Netherlands	47655,00	45562,74	42295,51
2006	(OST) Austria	24860,00	23794,78	24917,69
2006	(POL) Poland	7450,00	6147,25	6303,36
2006	(POR) Portugal	10003,00	9380,55	8952,82
2006	(SUO) Finland	18007,00	17678,12	20742,25
2006	(SVE) Sweden	8526,00	8440,17	14727,00
2006	(SVK) Slovakia	-112033,00	-98751,19	-34691,00
2006	(SVN) Slovenia	3209,00	3098,81	4898,19

2006	(UKI) United Kingdo	34516,00	32690,31	37564,83
2005	(BEL) Belgium	47865,00	46890,19	48135,82
2005	(CZE) Czech Republi	17940,00	16746,81	20685,18
2005	(DAN) Denmark	15586,00	15170,13	13692,34
2005	(DEU) Germany	29530,00	29409,69	30598,03
2005	(ELL) Greece	14076,00	13768,61	13090,49
2005	(ESP) Spain	20526,00	19718,25	22635,27
2005	(EST) Estonia	15026,00	14187,04	13381,86
2005	(FRA) France	29518,00	28959,96	30437,91
2005	(HUN) Hungary	5818,00	5586,14	6425,20
2005	(IRE) Ireland	18241,00	17688,96	17372,52
2005	(ITA) Italy	20900,00	20487,62	20354,73
2005	(LTU) Lithuania	7207,00	6742,03	6948,73
2005	(LUX) Luxembourg	37405,00	35900,98	35929,13
2005	(LVA) Latvia	10212,00	9596,11	9704,88
2005	(NED) Netherlands	39358,00	38591,43	38067,72
2005	(OST) Austria	23441,00	22861,55	23349,44
2005	(POL) Poland	5834,00	5047,97	5768,41
2005	(POR) Portugal	8354,00	8083,80	8550,45
2005	(SUO) Finland	19901,00	19717,89	19269,00
2005	(SVE) Sweden	11848,00	11972,91	8540,36
2005	(SVK) Slovakia	-12193,00	-11475,39	-38835,86
2005	(SVN) Slovenia	4989,00	4924,80	4694,20
2005	(UKI) United Kingdo	32672,00	31970,59	30803,30
2004	(BEL) Belgium	45864,00	45864,00	46377,09
2004	(CZE) Czech Republi	24303,00	24303,00	20524,90
2004	(DAN) Denmark	6299,00	6299,00	10734,56
2004	(DEU) Germany	29967,00	29967,00	29688,35
2004	(ELL) Greece	12209,00	12209,00	12988,80
2004	(ESP) Spain	25668,00	25668,00	22693,12
2004	(EST) Estonia	15722,00	15722,00	14954,52
2004	(FRA) France	30014,00	30014,00	29486,98
2004	(HUN) Hungary	6425,00	6425,00	6005,57
2004	(IRE) Ireland	17595,00	17595,00	17641,98
2004	(ITA) Italy	19966,00	19966,00	20226,81
2004	(LTU) Lithuania	7087,00	7087,00	6914,52
2004	(LUX) Luxembourg	36883,00	36883,00	36391,99
2004	(LVA) Latvia	8923,00	8923,00	9259,55
2004	(NED) Netherlands	30049,00	30049,00	34320,21
2004	(OST) Austria	23392,00	23392,00	23126,78
2004	(POL) Poland	6110,00	6110,00	5578,99
2004	(POR) Portugal	8187,00	8187,00	8135,40
2004	(SUO) Finland	20411,00	20411,00	20064,45
2004	(SVE) Sweden	5208,00	5208,00	8590,45
2004	(SVK) Slovakia	-6281,00	-6281,00	-8878,20
2004	(SVN) Slovenia	6059,00	6059,00	5491,90
2004	(UKI) United Kingdo	27749,00	27749,00	29859,79

Total assets (SE436)	totassets-ind	totasseindequali	indoutput	indinput	exchangerate
963854,00	768033,02	764379,59	1,26	1,25	1,00
747346,00	503595,54	513301,49	1,19	1,19	0,80
2688656,00	2130221,62	2109646,93	1,20	1,26	1,00
966056,00	811979,37	809122,49	1,19	1,19	1,00
112574,00	98277,31	97933,80	1,15	1,15	1,00
370015,00	311463,42	310905,87	1,19	1,19	1,00
350516,00	192145,59	184944,22	1,83	1,82	1,00
450417,00	381114,51	375777,73	1,18	1,18	1,00
206566,00	171890,70	169770,55	1,55	1,55	1,29
1013731,00	884965,49	879751,69	1,15	1,15	1,00
508683,00	415390,76	418054,18	1,22	1,22	1,00
134080,00	82611,45	81878,27	1,62	1,62	1,00
1239375,00	850762,86	838153,27	1,46	1,46	1,00
183227,00	105095,50	96914,06	1,85	1,85	1,06
3123033,00	2642303,90	2370978,55	1,18	1,18	1,00
580868,00	450000,67	420653,11	1,29	1,29	1,00
187180,00	134304,65	131599,93	1,32	1,32	0,95
101917,00	82067,75	82594,38	1,24	1,24	1,00
519812,00	411475,84	397887,15	1,26	1,26	1,00
1131414,00	891731,69	954167,38	1,27	1,27	1,00
1093310,00	721593,79	762655,24	1,17	1,14	0,75
226539,00	180734,52	182156,59	1,26	1,26	1,00
1979137,00	1504433,18	1710198,83	1,32	1,32	1,00
940830,00	760726,15	756024,34	1,24	1,24	1,00
748762,00	523007,43	513079,45	1,18	1,18	0,82
2597916,00	2089072,25	2096710,25	1,19	1,24	1,00
948330,00	806265,61	803478,70	1,18	1,18	1,00
110773,00	97590,30	101524,39	1,14	1,14	1,00
364523,00	310348,32	308247,14	1,17	1,17	1,00
313778,00	177742,86	180380,03	1,77	1,77	1,00
432991,00	370440,95	376508,71	1,17	1,17	1,00
206526,00	167650,40	170079,72	1,51	1,51	1,23
992697,00	874537,90	887859,23	1,14	1,14	1,00
508457,00	420717,60	411887,02	1,21	1,21	1,00
128089,00	81145,09	81773,14	1,58	1,58	1,00
1175701,00	825543,68	831037,62	1,42	1,42	1,00
151768,00	88732,62	94564,89	1,79	1,79	1,04
2454369,00	2099653,20	2264395,24	1,17	1,17	1,00
496975,00	391305,55	399410,63	1,27	1,27	1,00
178166,00	128895,21	129710,30	1,30	1,30	0,94
101789,00	83121,01	84301,13	1,22	1,22	1,00
478252,00	384298,47	395382,28	1,24	1,24	1,00
1202748,00	1016603,08	930142,93	1,25	1,25	1,06
1206970,00	803716,68	776739,88	1,15	1,13	0,75
226789,00	183578,65	178409,89	1,24	1,24	1,00
1910458,00	1915964,47	1783001,53	1,29	1,29	1,30
898902,00	739313,84	705613,99	1,22	1,22	1,00
696994,00	512635,37	511494,79	1,16	1,16	0,86

2544203,00	2070836,89	2065208,64	1,17	1,23	1,00
922114,00	792191,13	796311,51	1,16	1,16	1,00
122653,00	108705,56	104992,26	1,13	1,13	1,00
350956,00	302929,68	302494,25	1,16	1,16	1,00
291698,00	171251,63	174200,29	1,71	1,70	1,00
439761,00	377970,66	375793,00	1,16	1,16	1,00
201334,00	170698,07	165040,98	1,46	1,46	1,24
1014731,00	904074,31	889772,06	1,12	1,12	1,00
479576,00	399552,69	407086,06	1,20	1,20	1,00
123496,00	81562,88	79642,29	1,51	1,51	1,00
1143647,00	816806,31	833922,40	1,40	1,40	1,00
149256,00	89866,56	89848,44	1,74	1,74	1,04
2367913,00	2051228,62	2075193,68	1,15	1,15	1,00
448235,00	356925,68	368376,90	1,26	1,26	1,00
166982,00	125931,02	126027,28	1,28	1,28	0,96
105812,00	87714,62	86432,95	1,21	1,21	1,00
482211,00	390372,53	390566,66	1,24	1,24	1,00
1039633,00	882094,04	927080,11	1,22	1,22	1,04
1194156,00	804909,16	794370,83	1,14	1,12	0,75
207851,00	170916,50	177375,15	1,22	1,22	1,00
1978748,00	1928606,93	1909861,20	1,27	1,27	1,24
737113,00	616801,98	654612,47	1,20	1,20	1,00
669806,00	498841,58	494934,92	1,15	1,15	0,86
2490208,00	2035716,77	2045115,19	1,17	1,23	1,00
909388,00	790477,79	792394,24	1,15	1,15	1,00
122932,00	108680,94	110653,73	1,13	1,13	1,00
339756,00	294204,74	278123,21	1,15	1,15	1,00
290736,00	173606,37	173799,54	1,67	1,67	1,00
438601,00	378967,39	377130,47	1,16	1,16	1,00
184062,00	156774,48	161024,29	1,44	1,44	1,23
1002731,00	890703,97	917604,75	1,13	1,13	1,00
475893,00	400987,89	403702,42	1,19	1,19	1,00
113579,00	76218,89	79550,04	1,49	1,49	1,00
1194111,00	859417,21	848602,64	1,39	1,39	1,00
149775,00	90946,13	88038,59	1,72	1,72	1,04
2384353,00	2074699,21	2070664,31	1,15	1,15	1,00
440883,00	356899,46	357055,12	1,24	1,24	1,00
169941,00	123255,59	124046,97	1,27	1,27	0,92
104920,00	88463,22	88774,26	1,19	1,19	1,00
489483,00	397028,99	392602,28	1,23	1,23	1,00
1037127,00	882543,21	873170,18	1,20	1,20	1,03
1154951,00	774486,64	776782,66	1,15	1,12	0,75
214399,00	177630,29	175602,79	1,21	1,21	1,00
2028317,00	1885012,19	1877513,92	1,24	1,24	1,15
716722,00	607721,57	616293,33	1,18	1,18	1,00
623691,00	473327,82	530878,14	1,14	1,14	0,86
2472411,00	2028791,90	2054240,21	1,16	1,22	1,00
898461,00	794513,80	801898,80	1,13	1,13	1,00
130049,00	114574,69	105843,66	1,14	1,14	1,00
272487,00	237235,21	253681,44	1,15	1,15	1,00

292378,00	176540,63	175659,74	1,66	1,66	1,00
428530,00	374453,34	381386,50	1,14	1,14	1,00
178992,00	155600,31	156377,14	1,41	1,41	1,23
1000614,00	958035,97	907097,28	1,04	1,04	1,00
482789,00	410566,67	392004,83	1,18	1,18	1,00
120403,00	80868,36	78011,39	1,49	1,49	1,00
1206000,00	869584,39	875146,40	1,39	1,39	1,00
137188,00	83303,07	88883,13	1,72	1,72	1,04
2378846,00	2086065,09	2097293,92	1,14	1,14	1,00
431520,00	357340,21	366158,31	1,21	1,21	1,00
168234,00	122954,28	122640,09	1,26	1,26	0,92
104796,00	90144,94	92413,51	1,16	1,16	1,00
473465,00	390405,31	386219,71	1,21	1,21	1,00
1010858,00	854873,30	828170,94	1,18	1,18	1,00
1122351,00	750952,17	726996,44	1,15	1,13	0,75
213015,00	178261,59	171340,49	1,20	1,20	1,00
1945923,00	1818922,65	1834132,21	1,23	1,24	1,15
729126,00	624356,42	600267,46	1,17	1,17	1,00
843820,00	620465,01	553905,12	1,11	1,11	0,82
2530046,00	2098211,95	2066568,38	1,15	1,21	1,00
911109,00	820704,81	802433,04	1,11	1,11	1,00
109005,00	94275,36	100539,27	1,16	1,16	1,00
264312,00	229604,37	233820,77	1,15	1,15	1,00
284481,00	176832,22	172377,40	1,61	1,61	1,00
444631,00	390738,76	386347,11	1,14	1,14	1,00
180962,00	156756,62	154764,62	1,36	1,36	1,18
912340,00	872551,91	896875,16	1,05	1,05	1,00
424692,00	364459,93	378220,09	1,17	1,17	1,00
113525,00	76946,94	77958,92	1,48	1,48	1,00
1210108,00	896437,61	886359,11	1,35	1,35	1,00
149503,00	92400,19	87537,28	1,69	1,69	1,04
2424071,00	2131117,46	2112241,71	1,14	1,14	1,00
454113,00	384235,24	374446,65	1,18	1,18	1,00
164912,00	121710,39	119342,04	1,26	1,26	0,93
113855,00	98632,37	94717,41	1,15	1,15	1,00
443009,00	371224,83	377412,23	1,19	1,19	1,00
913082,00	747096,30	792813,19	1,16	1,16	0,95
981648,00	655550,52	653580,97	1,15	1,13	0,75
188106,00	158129,58	165247,22	1,19	1,19	1,00
1747485,00	1798461,80	1764129,73	1,21	1,21	1,25
655798,00	568724,38	579826,54	1,15	1,15	1,00
788751,00	567922,54	608008,54	1,09	1,09	0,79
2481823,00	2072701,27	2116015,97	1,14	1,20	1,00
862291,00	792080,52	787926,77	1,09	1,09	1,00
109847,00	92767,77	94357,30	1,18	1,18	1,00
268998,00	234622,73	244725,94	1,15	1,15	1,00
253223,00	163759,35	163035,13	1,55	1,55	1,00
444721,00	393849,23	388576,51	1,13	1,13	1,00
174813,00	151936,93	152463,32	1,32	1,32	1,15
888354,00	860037,59	834572,33	1,03	1,03	1,00

414339,00	359633,67	354413,00	1,15	1,15	1,00
110721,00	76061,47	77192,45	1,46	1,46	1,00
1185433,00	893055,32	877504,85	1,33	1,33	1,00
138347,00	86908,59	86455,54	1,66	1,66	1,04
2380444,00	2119542,59	2077114,70	1,12	1,12	1,00
443999,00	381764,49	376724,31	1,16	1,16	1,00
153893,00	113361,44	115494,89	1,25	1,25	0,92
107666,00	95374,93	94915,44	1,13	1,13	1,00
431248,00	370606,53	368659,79	1,16	1,16	1,00
934151,00	776469,97	751002,11	1,15	1,15	0,95
825687,00	554240,21	640233,27	1,15	1,12	0,75
186580,00	159350,49	159984,15	1,17	1,17	1,00
1663482,00	1675004,74	1702210,20	1,19	1,19	1,20
617896,00	546398,80	547483,00	1,13	1,13	1,00
887709,00	635638,06	593089,35	1,08	1,08	0,77
2544069,00	2177134,67	2164869,84	1,11	1,17	1,00
805491,00	750994,98	765009,09	1,07	1,07	1,00
114131,00	96028,77	88793,73	1,19	1,19	1,00
309857,00	269950,72	263110,22	1,15	1,15	1,00
220775,00	148513,83	149597,76	1,49	1,49	1,00
425420,00	381141,56	382262,90	1,12	1,12	1,00
171726,00	148696,41	143803,61	1,28	1,28	1,11
778714,00	771127,49	812120,82	1,01	1,01	1,00
384776,00	339145,39	336330,68	1,13	1,13	1,00
111284,00	78568,94	78651,21	1,42	1,42	1,00
1091214,00	843021,61	863916,58	1,29	1,29	1,00
121256,00	80057,83	80830,72	1,60	1,61	1,06
2192649,00	1980684,05	2042969,79	1,11	1,11	1,00
414982,00	364173,21	366545,49	1,14	1,14	1,00
149939,00	111412,83	111082,83	1,22	1,22	0,91
102838,00	90739,02	91109,25	1,13	1,13	1,00
411468,00	364148,01	365349,42	1,13	1,13	1,00
837958,00	729440,06	723639,31	1,14	1,14	0,99
1045757,00	710909,07	616261,17	1,13	1,11	0,75
189330,00	162472,37	165362,97	1,17	1,17	1,00
1499014,00	1633164,08	1617055,19	1,17	1,17	1,28
585714,00	527325,82	540127,02	1,11	1,11	1,00
781547,00	575707,46	590541,23	1,08	1,08	0,79
2607737,00	2244773,57	2281279,73	1,10	1,16	1,00
797912,00	751951,78	748171,24	1,06	1,06	1,00
91480,00	77584,63	84292,13	1,18	1,18	1,00
326912,00	284757,21	281250,26	1,15	1,15	1,00
192567,00	136520,11	149446,54	1,41	1,41	1,00
411073,00	371797,92	371407,73	1,11	1,11	1,00
149935,00	130777,48	133999,45	1,25	1,25	1,09
801095,00	805197,39	790094,73	1,00	0,99	1,00
346370,00	310212,98	313963,33	1,12	1,12	1,00
109306,00	81323,22	78652,39	1,34	1,34	1,00
1057038,00	855672,79	851298,36	1,24	1,24	1,00
107514,00	75525,74	77981,86	1,51	1,51	1,06

2241514,00	2028682,74	1951880,48	1,10	1,10	1,00
395791,00	353698,78	368947,55	1,12	1,12	1,00
146017,00	108474,22	109938,11	1,19	1,19	0,88
99106,00	87213,80	87823,60	1,14	1,14	1,00
397789,00	361293,71	354112,38	1,10	1,10	1,00
714624,00	665007,92	669560,45	1,12	1,12	1,05
844406,00	583634,23	608688,69	1,12	1,09	0,75
200989,00	174266,06	166546,23	1,16	1,16	1,00
1403393,00	1542996,75	1545211,96	1,15	1,15	1,26
595945,00	546656,42	532347,57	1,09	1,09	1,00
739401,00	560278,18	567137,92	1,09	1,09	0,83
2726002,00	2421930,96	2343890,78	1,10	1,13	1,00
781844,00	741566,97	734105,50	1,05	1,05	1,00
92832,00	79262,98	77962,47	1,17	1,17	1,00
331331,00	289042,84	290653,95	1,15	1,15	1,00
226229,00	163305,68	146761,33	1,39	1,39	1,00
395246,00	361283,71	365657,43	1,09	1,09	1,00
134772,00	122524,46	130733,75	1,22	1,22	1,11
815600,00	793959,29	824604,00	1,03	1,03	1,00
325217,00	292531,62	293133,38	1,11	1,11	1,00
99974,00	76065,01	76155,09	1,31	1,31	1,00
1019581,00	855200,68	851322,28	1,19	1,19	1,00
112004,00	78362,00	75285,56	1,51	1,51	1,06
2020928,00	1846274,65	1818459,78	1,09	1,09	1,00
431506,00	388970,65	373277,48	1,11	1,11	1,00
134130,00	109927,28	107722,52	1,17	1,17	0,96
96562,00	85517,96	85514,03	1,13	1,13	1,00
369750,00	336895,43	346677,24	1,10	1,10	1,00
587793,00	614233,36	624090,51	1,11	1,11	1,16
765295,00	531522,77	589590,69	1,12	1,08	0,75
189832,00	162900,27	180448,81	1,17	1,17	1,00
1264592,00	1459475,04	1432667,58	1,13	1,13	1,30
567223,00	523060,46	524494,34	1,08	1,08	1,00
768040,00	565428,14	568052,28	1,06	1,06	0,78
2644430,00	2364967,81	2332215,48	1,09	1,12	1,00
733770,00	708797,76	720631,25	1,04	1,04	1,00
87970,00	77039,79	78945,25	1,14	1,14	1,00
341294,00	298161,80	290096,75	1,14	1,14	1,00
194911,00	140458,20	144845,55	1,39	1,39	1,00
397814,00	363890,68	360280,34	1,09	1,09	1,00
163289,00	138899,31	129686,98	1,18	1,18	1,00
941425,00	874655,32	872993,53	1,08	1,08	1,00
302483,00	276655,53	290090,57	1,09	1,09	1,00
96602,00	71077,04	73210,52	1,36	1,36	1,00
991281,00	843093,37	849857,77	1,18	1,18	1,00
115589,00	71968,95	73238,70	1,68	1,68	1,04
1726105,00	1580421,94	1656859,49	1,09	1,09	1,00
410625,00	377163,00	382029,04	1,09	1,09	1,00
151546,47	104766,06	106040,81	1,12	1,12	0,78
93603,00	83810,32	85585,28	1,12	1,12	1,00

368622,00	341842,58	339349,26	1,08	1,08	1,00
612165,00	593030,26	590494,79	1,09	1,09	1,05
848782,00	653615,07	732886,55	1,10	1,10	0,85
230114,00	204180,12	177224,81	1,13	1,13	1,00
1192532,00	1295530,95	1328493,00	1,11	1,11	1,21
536077,00	503766,13	488446,50	1,06	1,06	1,00
692339,00	578450,53	590156,42	1,04	1,04	0,87
2374516,20	2209747,67	2356919,39	1,08	1,08	1,00
729987,00	711529,02	706905,33	1,03	1,03	1,00
88130,00	80532,97	79795,78	1,09	1,09	1,00
316880,00	283085,62	279141,67	1,12	1,12	1,00
169748,00	130772,76	128068,74	1,30	1,30	1,00
379840,00	355666,64	352881,16	1,07	1,07	1,00
143419,00	127637,18	124640,32	1,12	1,12	1,00
1026134,00	950365,97	903686,21	1,08	1,08	1,00
321485,00	301084,57	296493,19	1,07	1,07	1,00
89807,00	72489,52	64105,83	1,24	1,24	1,00
963521,00	851279,25	836481,25	1,13	1,13	1,00
99738,00	69385,14	69548,85	1,50	1,50	1,04
1648284,00	1543881,87	1523287,91	1,07	1,07	1,00
405737,00	379953,46	373306,44	1,07	1,07	1,00
133776,10	103429,09	102327,37	1,08	1,08	0,84
95977,00	87427,55	85284,38	1,10	1,10	1,00
355115,00	339309,77	329611,79	1,05	1,05	1,00
586439,00	564220,76	576830,30	1,05	1,05	1,01
1279630,00	1013521,80	923193,99	1,07	1,07	0,85
177564,00	164594,04	177762,39	1,08	1,08	1,00
1295249,00	1230473,01	1177088,49	1,08	1,08	1,03
457783,00	438512,92	455311,14	1,04	1,04	1,00
711669,00	626590,58	601249,40	1,01	1,01	0,89
2615415,51	2496042,69	2331627,71	1,05	1,05	1,00
706066,00	700389,19	702314,14	1,01	1,01	1,00
86564,00	81814,59	80646,96	1,06	1,06	1,00
277282,00	256177,60	252967,52	1,08	1,08	1,00
130449,00	112975,27	121873,79	1,15	1,15	1,00
353108,00	339086,14	343223,77	1,04	1,04	1,00
108826,00	107384,46	117447,44	1,06	1,06	1,05
945225,00	886037,34	851045,80	1,07	1,07	1,00
324805,00	311739,47	309647,56	1,04	1,04	1,00
55628,00	48750,94	55541,82	1,14	1,14	1,00
908950,00	815071,14	838137,58	1,12	1,12	1,00
80524,00	67292,46	67936,97	1,25	1,25	1,04
1511920,00	1445559,93	1491620,36	1,05	1,05	1,00
379005,00	362802,85	365608,03	1,04	1,04	1,00
119720,56	98786,96	100502,77	1,04	1,04	0,86
90214,00	84615,26	83769,40	1,07	1,07	1,00
313388,00	307683,02	311891,63	1,02	1,02	1,00
579048,00	573239,88	556807,36	1,02	1,02	1,01
1250754,00	1102445,08	1233022,19	1,06	1,06	0,93
170358,00	164513,00	169128,48	1,04	1,04	1,00

1061437,00	1005261,52	1062541,56	1,05	1,05	1,00
432476,00	423654,37	423410,43	1,02	1,02	1,00
641357,00	598707,09	600603,56	1,00	1,00	0,93
2352005,60	2289092,77	2312214,73	1,03	1,03	1,00
697903,00	695024,20	697828,80	1,00	1,00	1,00
81373,00	79593,30	79902,30	1,02	1,02	1,00
228643,00	219639,32	231146,64	1,04	1,04	1,00
129078,00	121873,34	118277,54	1,06	1,06	1,00
341408,00	334918,51	337556,55	1,02	1,02	1,00
122194,00	117320,68	115616,05	1,03	1,03	0,99
739080,00	716734,11	720635,81	1,03	1,03	1,00
322485,00	316118,63	304062,70	1,02	1,02	1,00
48517,00	45385,00	45848,98	1,07	1,07	1,00
883635,00	848062,35	834793,50	1,04	1,04	1,00
71438,00	67133,30	65040,58	1,11	1,11	1,04
1514835,00	1485419,28	1456494,40	1,02	1,02	1,00
363016,00	354067,77	356076,21	1,03	1,03	1,00
114762,65	99292,27	100343,76	1,03	1,03	0,89
81907,00	79265,38	82840,55	1,03	1,03	1,00
291353,00	288682,10	289370,04	1,01	1,01	1,00
527412,00	532961,45	516253,77	1,01	1,01	1,02
1682114,00	1583099,68	1431379,92	1,03	1,03	0,97
180607,00	178278,40	175412,80	1,02	1,02	1,00
972826,00	951890,14	940259,22	1,03	1,03	1,00
408064,00	408064,00	415859,19	1,00	1,00	1,00
576513,00	576513,00	587610,04	1,00	1,00	1,00
2151508,74	2151508,74	2220300,76	1,00	1,00	1,00
698073,00	698073,00	696548,60	1,00	1,00	1,00
78299,00	78299,00	78946,15	1,00	1,00	1,00
217623,00	217623,00	218631,16	1,00	1,00	1,00
119984,00	119984,00	120928,67	1,00	1,00	1,00
338665,00	338665,00	336791,76	1,00	1,00	1,00
122143,00	122143,00	119731,84	1,00	1,00	1,00
559136,00	559136,00	637935,05	1,00	1,00	1,00
284330,00	284330,00	300224,31	1,00	1,00	1,00
43411,00	43411,00	44398,00	1,00	1,00	1,00
841247,00	841247,00	844654,68	1,00	1,00	1,00
60696,00	60696,00	63914,65	1,00	1,00	1,00
1438504,00	1438504,00	1461961,64	1,00	1,00	1,00
351358,00	351358,00	352712,88	1,00	1,00	1,00
102952,06	102952,06	101122,17	1,00	1,00	1,00
84641,00	84641,00	81953,19	1,00	1,00	1,00
271745,00	271745,00	280213,55	1,00	1,00	1,00
442560,00	442560,00	487760,72	1,00	1,00	1,00
1608595,00	1608595,00	1595847,34	1,00	1,00	1,00
183447,00	183447,00	180862,70	1,00	1,00	1,00
863626,00	863626,00	907758,07	1,00	1,00	1,00
