# Supplementary material for: The relationship between income and assets in farms and context of sustainable development
Source: PLoS One. 2022 Mar 14;17(3):e0265128. doi: 10.1371/journal.pone.0265128 (PMC8920205; doi:10.1371/journal.pone.0265128)
Supplement: S2 Table — Source: Own calculation based on data of the FADN system. (PDF) [file pone.0265128.s002.pdf]

S2 Table. Variables used to the panel models

Year	Country	Total Utilised A	Arable land	Cereals (SE	sharecereals	Sust-sharecer	Total livestock
2018	(BEL) Belgium	51,94	32,58	11,70	0,36	1,00	136,23
2018	(CZE) Czech Republi	192,24	137,74	75,06	0,54	1,00	94,12
2018	(DEU) Germany	91,28	66,80	35,72	0,53	1,00	89,98
2018	(ELL) Greece	9,62	5,46	2,30	0,42	1,00	5,90
2018	(ESP) Spain	46,45	24,83	14,05	0,57	1,00	32,86
2018	(EST) Estonia	140,00	101,25	53,98	0,53	1,00	36,90
2018	(FRA) France	88,10	62,20	31,18	0,50	1,00	74,26
2018	(HUN) Hungary	44,84	36,77	21,22	0,58	1,00	17,80
2018	(IRE) Ireland	48,76	4,50	3,72	0,83	0,00	65,03
2018	(ITA) Italy	21,58	13,47	5,90	0,44	1,00	19,53
2018	(LTU) Lithuania	49,48	37,24	21,47	0,58	1,00	11,85
2018	(LUX) Luxembourg	85,68	41,72	17,03	0,41	1,00	124,25
2018	(LVA) Latvia	66,13	52,49	27,48	0,52	1,00	23,25
2018	(NED) Netherlands	39,30	22,08	3,69	0,17	1,00	145,57
2018	(OST) Austria	33,19	19,06	10,20	0,54	1,00	30,10
2018	(POL) Poland	19,64	15,93	10,86	0,68	0,00	12,00
2018	(POR) Portugal	22,53	8,31	1,70	0,20	1,00	15,05
2018	(SUO) Finland	67,24	60,15	30,18	0,50	1,00	29,96
2018	(SVE) Sweden	106,58	91,57	39,96	0,44	1,00	59,73
2018	(SVN) Slovenia	10,47	3,77	2,16	0,57	1,00	10,75
2018	(UKI) United Kingdo	158,56	63,06	32,41	0,51	1,00	130,13
2017	(BEL) Belgium	50,11	30,86	11,49	0,37	1,00	138,03
2017	(CZE) Czech Republi	205,77	148,97	82,21	0,55	1,00	93,67
2017	(DEU) Germany	89,84	65,92	36,08	0,55	1,00	91,00
2017	(ELL) Greece	10,12	5,53	2,62	0,47	1,00	5,90
2017	(ESP) Spain	48,76	26,03	14,61	0,56	1,00	33,05
2017	(EST) Estonia	128,63	91,69	45,83	0,50	1,00	39,39
2017	(FRA) France	86,68	61,10	31,27	0,51	1,00	72,53
2017	(HUN) Hungary	47,03	39,27	22,86	0,58	1,00	18,15
2017	(IRE) Ireland	48,53	4,63	3,87	0,84	0,00	65,44
2017	(ITA) Italy	20,38	12,60	5,54	0,44	1,00	19,12
2017	(LTU) Lithuania	47,93	35,94	19,84	0,55	1,00	12,50
2017	(LUX) Luxembourg	83,11	40,08	17,41	0,43	1,00	115,11
2017	(LVA) Latvia	62,01	47,54	25,30	0,53	1,00	19,94
2017	(NED) Netherlands	35,61	20,29	3,62	0,18	1,00	133,09
2017	(OST) Austria	32,46	18,57	9,94	0,54	1,00	30,33
2017	(POL) Poland	18,95	15,25	10,09	0,66	0,00	12,27
2017	(POR) Portugal	24,15	10,15	1,99	0,20	1,00	14,96
2017	(SUO) Finland	60,79	54,83	28,24	0,52	1,00	27,75
2017	(SVE) Sweden	108,68	94,02	41,18	0,44	1,00	71,13
2017	(SVN) Slovenia	9,84	3,30	1,86	0,56	1,00	10,38
2017	(UKI) United Kingdo	158,12	65,08	34,35	0,53	1,00	140,00
2016	(BEL) Belgium	49,92	30,88	12,35	0,40	1,00	133,54
2016	(CZE) Czech Republi	204,63	148,72	82,90	0,56	1,00	92,54
2016	(DEU) Germany	87,62	64,06	34,99	0,55	1,00	89,80
2016	(ELL) Greece	10,38	5,50	2,77	0,50	1,00	6,14
2016	(ESP) Spain	47,95	26,20	14,92	0,57	1,00	32,87
2016	(EST) Estonia	127,32	91,55	48,18	0,53	1,00	37,82

2016	(FRA) France	86,85	61,34	31,41	0,51	1,00	73,53
2016	(HUN) Hungary	47,93	40,50	24,98	0,62	1,00	17,55
2016	(IRE) Ireland	48,38	4,86	4,14	0,85	0,00	63,47
2016	(ITA) Italy	20,58	12,71	5,97	0,47	1,00	18,63
2016	(LTU) Lithuania	47,94	38,16	20,46	0,54	1,00	12,22
2016	(LUX) Luxembourg	81,64	39,94	17,43	0,44	1,00	111,09
2016	(LVA) Latvia	63,43	47,32	26,03	0,55	1,00	19,94
2016	(NED) Netherlands	36,02	20,33	3,95	0,19	1,00	136,80
2016	(OST) Austria	28,23	15,45	8,39	0,54	1,00	24,65
2016	(POL) Poland	18,78	15,16	10,18	0,67	0,00	12,04
2016	(POR) Portugal	23,96	10,24	2,12	0,21	1,00	14,79
2016	(SUO) Finland	60,11	53,51	28,89	0,54	1,00	29,54
2016	(SVE) Sweden	109,49	94,82	43,33	0,46	1,00	75,36
2016	(SVN) Slovenia	9,78	3,31	1,88	0,57	1,00	10,46
2016	(UKI) United Kingdo	161,67	65,33	34,22	0,52	1,00	139,08
2015	(BEL) Belgium	51,19	30,91	13,35	0,43	1,00	128,82
2015	(CZE) Czech Republi	203,93	148,19	84,35	0,57	1,00	91,41
2015	(DEU) Germany	88,44	64,99	36,22	0,56	1,00	89,50
2015	(ELL) Greece	10,02	5,42	2,76	0,51	1,00	6,00
2015	(ESP) Spain	47,73	25,99	14,20	0,55	1,00	31,66
2015	(EST) Estonia	127,19	90,44	48,65	0,54	1,00	39,62
2015	(FRA) France	86,55	61,56	31,68	0,51	1,00	72,51
2015	(HUN) Hungary	49,93	41,61	25,09	0,60	1,00	20,80
2015	(IRE) Ireland	49,66	5,44	3,66	0,67	0,00	59,28
2015	(ITA) Italy	20,42	12,55	5,72	0,46	1,00	18,53
2015	(LTU) Lithuania	46,33	37,32	21,11	0,57	1,00	11,98
2015	(LUX) Luxembourg	82,80	41,27	19,02	0,46	1,00	112,73
2015	(LVA) Latvia	64,19	48,20	26,21	0,54	1,00	21,54
2015	(NED) Netherlands	36,27	20,78	4,50	0,22	1,00	135,16
2015	(OST) Austria	28,69	15,46	8,32	0,54	1,00	24,45
2015	(POL) Poland	18,46	14,72	9,71	0,66	1,00	12,09
2015	(POR) Portugal	24,66	11,19	2,12	0,19	1,00	14,32
2015	(SUO) Finland	61,34	54,81	30,23	0,55	1,00	30,06
2015	(SVE) Sweden	108,91	94,31	43,38	0,46	1,00	76,15
2015	(SVN) Slovenia	9,77	3,22	1,91	0,59	1,00	9,96
2015	(UKI) United Kingdo	157,94	64,66	33,78	0,52	1,00	135,48
2014	(BEL) Belgium	51,17	31,47	12,86	0,41	1,00	124,87
2014	(CZE) Czech Republi	201,75	145,44	84,38	0,58	1,00	88,68
2014	(DEU) Germany	88,59	65,47	35,97	0,55	1,00	91,48
2014	(ELL) Greece	10,00	5,59	2,98	0,53	1,00	6,02
2014	(ESP) Spain	40,87	20,94	12,30	0,59	1,00	21,88
2014	(EST) Estonia	128,72	93,26	49,15	0,53	1,00	41,68
2014	(FRA) France	86,58	62,55	31,93	0,51	1,00	71,91
2014	(HUN) Hungary	49,22	41,79	27,18	0,65	1,00	18,64
2014	(IRE) Ireland	50,06	5,70	3,81	0,67	0,00	59,59
2014	(ITA) Italy	20,50	12,54	5,95	0,47	1,00	18,01
2014	(LTU) Lithuania	47,48	40,61	22,37	0,55	1,00	12,65
2014	(LUX) Luxembourg	82,03	41,10	18,61	0,45	1,00	111,05
2014	(LVA) Latvia	65,07	48,38	26,88	0,56	1,00	21,32
2014	(NED) Netherlands	36,86	21,17	4,27	0,20	1,00	137,71

2014	(OST) Austria	28,63	15,60	8,46	0,54	1,00	24,04
2014	(POL) Poland	18,40	14,62	9,98	0,68	0,00	12,07
2014	(POR) Portugal	26,83	11,85	2,53	0,21	1,00	14,99
2014	(SUO) Finland	58,41	52,52	29,68	0,57	1,00	30,23
2014	(SVE) Sweden	106,77	93,35	44,53	0,48	1,00	70,86
2014	(SVN) Slovenia	9,80	3,59	2,15	0,60	1,00	9,90
2014	(UKI) United Kingdo	163,84	62,54	32,60	0,52	1,00	128,80
2013	(BEL) Belgium	50,98	31,71	13,22	0,42	1,00	127,48
2013	(CZE) Czech Republi	201,99	142,40	82,07	0,58	1,00	88,71
2013	(DEU) Germany	88,90	65,79	36,33	0,55	1,00	90,50
2013	(ELL) Greece	9,81	5,64	2,90	0,51	1,00	5,82
2013	(ESP) Spain	40,95	20,94	12,70	0,61	1,00	23,40
2013	(EST) Estonia	136,95	98,62	50,19	0,51	1,00	40,41
2013	(FRA) France	86,44	61,95	31,68	0,51	1,00	71,26
2013	(HUN) Hungary	48,72	41,23	26,76	0,65	1,00	17,27
2013	(IRE) Ireland	49,51	6,26	4,11	0,66	1,00	57,98
2013	(ITA) Italy	17,01	11,60	5,11	0,44	1,00	15,58
2013	(LTU) Lithuania	48,55	41,61	21,64	0,52	1,00	12,66
2013	(LUX) Luxembourg	82,90	41,31	18,68	0,45	1,00	111,84
2013	(LVA) Latvia	69,09	51,46	26,12	0,51	1,00	20,59
2013	(NED) Netherlands	36,46	16,78	3,98	0,24	1,00	137,16
2013	(OST) Austria	32,71	15,28	9,07	0,59	1,00	24,62
2013	(POL) Poland	18,81	15,02	10,36	0,69	0,00	11,62
2013	(POR) Portugal	26,35	12,78	2,16	0,17	1,00	14,23
2013	(SUO) Finland	57,71	51,77	28,82	0,56	1,00	27,07
2013	(SVE) Sweden	102,77	89,72	39,39	0,44	1,00	70,29
2013	(SVN) Slovenia	10,56	4,21	2,56	0,61	1,00	10,83
2013	(UKI) United Kingdo	164,49	64,01	32,36	0,51	1,00	125,01
2012	(BEL) Belgium	50,39	31,15	13,05	0,42	1,00	125,96
2012	(CZE) Czech Republi	199,16	138,66	83,02	0,60	1,00	87,15
2012	(DEU) Germany	87,89	65,31	36,49	0,56	1,00	92,11
2012	(ELL) Greece	9,53	5,47	3,01	0,55	1,00	5,90
2012	(ESP) Spain	40,04	21,10	12,65	0,60	1,00	22,48
2012	(EST) Estonia	134,05	98,90	50,27	0,51	1,00	38,57
2012	(FRA) France	86,84	62,68	31,64	0,50	1,00	71,86
2012	(HUN) Hungary	48,45	40,83	27,06	0,66	0,00	16,70
2012	(IRE) Ireland	49,26	6,08	4,02	0,66	0,00	58,68
2012	(ITA) Italy	16,62	11,32	5,21	0,46	1,00	14,92
2012	(LTU) Lithuania	46,38	40,59	21,02	0,52	1,00	11,87
2012	(LUX) Luxembourg	83,88	42,56	18,49	0,43	1,00	109,05
2012	(LVA) Latvia	70,73	51,47	25,56	0,50	1,00	19,38
2012	(NED) Netherlands	37,56	17,06	4,24	0,25	1,00	138,77
2012	(OST) Austria	31,57	15,24	9,16	0,60	1,00	24,47
2012	(POL) Poland	18,67	14,92	10,64	0,71	0,00	11,66
2012	(POR) Portugal	26,54	13,03	2,56	0,20	1,00	14,67
2012	(SUO) Finland	56,87	51,00	27,72	0,54	1,00	26,57
2012	(SVE) Sweden	101,63	88,48	40,81	0,46	1,00	70,56
2012	(SVN) Slovenia	10,93	4,42	2,71	0,61	1,00	11,15
2012	(UKI) United Kingdo	157,30	61,95	31,53	0,51	1,00	126,81
2011	(BEL) Belgium	48,41	28,36	11,85	0,42	1,00	128,54

2011	(CZE) Czech Republi	228,79	162,96	98,40	0,60	1,00	103,63
2011	(DEU) Germany	84,84	62,23	34,40	0,55	1,00	86,19
2011	(ELL) Greece	9,06	5,00	2,70	0,54	1,00	6,46
2011	(ESP) Spain	36,50	20,03	11,36	0,57	1,00	24,36
2011	(EST) Estonia	125,49	93,82	45,52	0,49	1,00	36,48
2011	(FRA) France	87,68	62,91	30,84	0,49	1,00	73,71
2011	(HUN) Hungary	52,69	43,72	26,70	0,61	1,00	18,92
2011	(IRE) Ireland	43,37	5,39	3,37	0,63	1,00	48,14
2011	(ITA) Italy	15,90	10,90	4,80	0,44	1,00	12,13
2011	(LTU) Lithuania	46,68	41,69	21,14	0,51	1,00	13,11
2011	(LUX) Luxembourg	78,47	40,33	17,56	0,44	1,00	102,75
2011	(LVA) Latvia	71,75	51,25	24,42	0,48	1,00	19,90
2011	(NED) Netherlands	36,67	17,13	3,88	0,23	1,00	130,08
2011	(OST) Austria	30,71	15,17	9,06	0,60	1,00	24,73
2011	(POL) Poland	18,63	15,11	10,77	0,71	0,00	13,11
2011	(POR) Portugal	25,12	12,88	2,19	0,17	1,00	14,59
2011	(SUO) Finland	54,36	48,34	24,89	0,51	1,00	28,58
2011	(SVE) Sweden	98,86	84,32	37,20	0,44	1,00	62,69
2011	(SVN) Slovenia	11,13	4,47	2,62	0,59	1,00	12,60
2011	(UKI) United Kingdo	155,56	64,57	33,11	0,51	1,00	129,23
2010	(BEL) Belgium	48,37	28,38	12,26	0,43	1,00	130,08
2010	(CZE) Czech Republi	224,50	162,05	96,16	0,59	1,00	101,16
2010	(DEU) Germany	85,54	63,23	35,13	0,56	1,00	86,20
2010	(ELL) Greece	8,46	5,30	2,96	0,56	1,00	6,70
2010	(ESP) Spain	36,03	19,73	11,53	0,58	1,00	24,44
2010	(EST) Estonia	122,42	88,83	41,25	0,46	1,00	36,27
2010	(FRA) France	87,20	62,72	30,74	0,49	1,00	72,02
2010	(HUN) Hungary	51,46	42,82	26,68	0,62	1,00	18,43
2010	(IRE) Ireland	44,08	5,50	3,14	0,57	1,00	48,65
2010	(ITA) Italy	15,88	11,10	5,00	0,45	1,00	11,75
2010	(LTU) Lithuania	47,85	43,75	21,90	0,50	1,00	13,30
2010	(LUX) Luxembourg	78,47	41,29	17,90	0,43	1,00	101,00
2010	(LVA) Latvia	70,29	52,22	25,42	0,49	1,00	19,87
2010	(NED) Netherlands	36,35	16,91	3,80	0,22	1,00	131,44
2010	(OST) Austria	31,01	15,13	9,08	0,60	1,00	24,46
2010	(POL) Poland	18,48	15,02	10,52	0,70	0,00	13,05
2010	(POR) Portugal	24,61	12,22	2,05	0,17	1,00	12,95
2010	(SUO) Finland	55,12	49,95	23,39	0,47	1,00	28,61
2010	(SVE) Sweden	98,61	83,80	35,14	0,42	1,00	65,26
2010	(SVN) Slovenia	11,40	4,02	2,17	0,54	1,00	12,52
2010	(UKI) United Kingdo	157,79	64,68	33,20	0,51	1,00	129,90
2009	(BEL) Belgium	47,40	28,55	12,25	0,43	1,00	123,48
2009	(CZE) Czech Republi	226,07	161,55	97,12	0,60	1,00	103,08
2009	(DEU) Germany	85,40	63,67	36,28	0,57	1,00	89,14
2009	(ELL) Greece	8,20	5,25	3,11	0,59	1,00	6,68
2009	(ESP) Spain	36,35	20,13	11,55	0,57	1,00	23,15
2009	(EST) Estonia	134,05	101,80	52,19	0,51	1,00	41,22
2009	(FRA) France	86,12	62,04	31,46	0,51	1,00	72,17
2009	(HUN) Hungary	49,69	40,94	26,55	0,65	1,00	17,85
2009	(IRE) Ireland	43,82	5,63	3,11	0,55	1,00	48,97

2009	(ITA) Italy	16,58	11,71	5,41	0,46	1,00	13,71
2009	(LTU) Lithuania	46,52	40,93	22,29	0,54	1,00	13,67
2009	(LUX) Luxembourg	80,06	42,12	19,54	0,46	1,00	101,10
2009	(LVA) Latvia	71,06	51,65	25,84	0,50	1,00	22,86
2009	(NED) Netherlands	35,70	15,91	3,70	0,23	1,00	130,39
2009	(OST) Austria	32,18	16,26	9,91	0,61	1,00	26,97
2009	(POL) Poland	18,42	15,03	10,84	0,72	0,00	13,24
2009	(POR) Portugal	25,68	14,36	2,35	0,16	1,00	13,49
2009	(SUO) Finland	53,06	51,65	26,06	0,50	1,00	27,77
2009	(SVE) Sweden	95,97	80,81	36,77	0,46	1,00	63,60
2009	(SVN) Slovenia	10,87	4,22	2,43	0,58	1,00	12,37
2009	(UKI) United Kingdo	159,02	66,80	34,30	0,51	1,00	130,63
2008	(BEL) Belgium	44,08	26,93	11,48	0,43	1,00	111,87
2008	(CZE) Czech Republi	224,91	160,29	97,42	0,61	1,00	118,30
2008	(DEU) Germany	77,74	57,31	33,46	0,58	1,00	83,28
2008	(ELL) Greece	7,53	4,96	2,89	0,58	1,00	6,25
2008	(ESP) Spain	37,33	20,61	13,03	0,63	1,00	24,06
2008	(EST) Estonia	112,88	86,36	43,10	0,50	1,00	34,89
2008	(FRA) France	84,54	59,98	30,42	0,51	1,00	73,38
2008	(HUN) Hungary	56,07	46,63	30,66	0,66	1,00	19,95
2008	(IRE) Ireland	45,90	5,69	2,97	0,52	1,00	51,78
2008	(ITA) Italy	14,80	10,41	5,50	0,53	1,00	12,63
2008	(LTU) Lithuania	43,51	38,18	20,65	0,54	1,00	15,10
2008	(LUX) Luxembourg	76,32	39,23	18,77	0,48	1,00	100,10
2008	(LVA) Latvia	67,73	48,99	24,78	0,51	1,00	23,60
2008	(NED) Netherlands	34,74	15,56	3,62	0,23	1,00	122,78
2008	(OST) Austria	31,48	16,05	9,84	0,61	1,00	26,07
2008	(POL) Poland	19,62	16,28	11,88	0,73	0,00	13,40
2008	(POR) Portugal	25,50	14,92	2,45	0,16	1,00	14,42
2008	(SUO) Finland	52,22	51,02	26,23	0,51	1,00	28,43
2008	(SVE) Sweden	90,38	76,07	34,44	0,45	1,00	62,29
2008	(SVN) Slovenia	10,81	3,82	2,19	0,57	1,00	12,67
2008	(UKI) United Kingdo	150,91	61,28	33,00	0,54	1,00	125,74
2007	(BEL) Belgium	43,21	25,59	10,21	0,40	1,00	112,28
2007	(CZE) Czech Republi	222,53	161,10	99,71	0,62	1,00	116,23
2007	(DEU) Germany	78,41	57,73	32,19	0,56	1,00	82,81
2007	(ELL) Greece	7,56	4,97	2,75	0,55	1,00	6,68
2007	(ESP) Spain	36,27	20,43	12,51	0,61	1,00	21,34
2007	(EST) Estonia	109,25	91,39	40,96	0,45	1,00	34,34
2007	(FRA) France	84,73	60,57	28,98	0,48	1,00	72,45
2007	(HUN) Hungary	54,42	45,32	29,81	0,66	1,00	20,80
2007	(IRE) Ireland	45,89	6,04	2,73	0,45	1,00	52,23
2007	(ITA) Italy	14,77	11,05	5,25	0,48	1,00	10,84
2007	(LTU) Lithuania	43,93	38,13	19,46	0,51	1,00	15,87
2007	(LUX) Luxembourg	76,53	39,54	17,59	0,44	1,00	94,67
2007	(LVA) Latvia	68,60	49,50	24,38	0,49	1,00	23,78
2007	(NED) Netherlands	34,48	15,41	3,35	0,22	1,00	121,08
2007	(OST) Austria	30,98	16,42	9,76	0,59	1,00	24,91
2007	(POL) Poland	18,33	15,19	10,78	0,71	0,00	13,15
2007	(POR) Portugal	25,21	15,74	2,09	0,13	1,00	15,29

2007	(SUO) Finland	51,39	49,84	24,23	0,49	1,00	28,20
2007	(SVE) Sweden	90,67	76,48	31,77	0,42	1,00	57,70
2007	(SVN) Slovenia	10,84	3,68	1,97	0,54	1,00	12,72
2007	(UKI) United Kingdo	154,52	64,70	31,34	0,48	1,00	127,57
2006	(BEL) Belgium	41,55	24,43	9,75	0,40	1,00	105,82
2006	(CZE) Czech Republi	227,94	164,03	98,87	0,60	1,00	123,20
2006	(DEU) Germany	77,15	56,67	31,27	0,55	1,00	80,32
2006	(ELL) Greece	7,85	5,16	2,86	0,55	1,00	6,41
2006	(ESP) Spain	35,33	20,48	12,48	0,61	1,00	19,92
2006	(EST) Estonia	96,24	74,37	34,34	0,46	1,00	30,01
2006	(FRA) France	81,49	58,44	27,86	0,48	1,00	69,94
2006	(HUN) Hungary	46,19	38,39	24,97	0,65	1,00	17,35
2006	(IRE) Ireland	44,54	7,09	3,28	0,46	1,00	51,33
2006	(ITA) Italy	16,17	12,40	5,11	0,41	1,00	12,54
2006	(LTU) Lithuania	28,87	23,47	12,04	0,51	1,00	11,39
2006	(LUX) Luxembourg	74,86	38,28	16,66	0,44	1,00	89,78
2006	(LVA) Latvia	58,39	41,11	19,11	0,46	1,00	21,09
2006	(NED) Netherlands	31,80	14,55	3,01	0,21	1,00	107,08
2006	(OST) Austria	30,64	15,85	8,81	0,56	1,00	24,24
2006	(POL) Poland	17,35	14,39	10,35	0,72	0,00	12,83
2006	(POR) Portugal	24,53	14,95	2,61	0,17	1,00	13,04
2006	(SUO) Finland	50,62	49,75	23,30	0,47	1,00	26,87
2006	(SVE) Sweden	88,81	71,90	28,58	0,40	1,00	60,82
2006	(SVN) Slovenia	10,17	3,27	1,60	0,49	1,00	11,85
2006	(UKI) United Kingdo	147,58	62,47	28,95	0,46	1,00	122,57
2005	(BEL) Belgium	41,12	23,90	9,48	0,40	1,00	107,15
2005	(CZE) Czech Republi	230,95	169,10	102,40	0,61	1,00	127,67
2005	(DEU) Germany	77,68	57,88	30,66	0,53	1,00	78,89
2005	(ELL) Greece	7,98	5,16	3,08	0,60	1,00	6,11
2005	(ESP) Spain	33,72	19,43	11,59	0,60	1,00	19,20
2005	(EST) Estonia	104,56	79,96	37,77	0,47	1,00	33,97
2005	(FRA) France	80,81	58,19	28,05	0,48	1,00	69,15
2005	(HUN) Hungary	49,04	40,75	27,25	0,67	0,00	20,85
2005	(IRE) Ireland	41,61	6,26	2,53	0,40	1,00	51,50
2005	(ITA) Italy	15,66	12,04	5,35	0,44	1,00	12,65
2005	(LTU) Lithuania	30,45	24,23	12,19	0,50	1,00	9,96
2005	(LUX) Luxembourg	71,74	35,49	15,78	0,44	1,00	88,30
2005	(LVA) Latvia	56,09	41,24	19,06	0,46	1,00	20,95
2005	(NED) Netherlands	30,78	15,09	2,88	0,19	1,00	106,11
2005	(OST) Austria	31,63	16,80	9,71	0,58	1,00	24,99
2005	(POL) Poland	17,18	15,64	10,36	0,66	0,00	12,57
2005	(POR) Portugal	23,64	14,50	2,65	0,18	1,00	13,32
2005	(SUO) Finland	48,12	47,86	23,16	0,48	1,00	26,96
2005	(SVE) Sweden	87,38	71,30	28,85	0,40	1,00	54,65
2005	(SVN) Slovenia	10,53	3,36	1,67	0,50	1,00	12,07
2005	(UKI) United Kingdo	144,24	61,87	29,05	0,47	1,00	121,79
2004	(BEL) Belgium	40,79	24,12	8,83	0,37	1,00	106,18
2004	(CZE) Czech Republi	227,94	167,74	101,95	0,61	1,00	131,36
2004	(DEU) Germany	74,75	55,02	31,25	0,57	1,00	79,93
2004	(ELL) Greece	7,46	4,99	3,01	0,60	1,00	5,95

2004	(ESP) Spain	34,06	18,89	11,54	0,61	1,00	20,43
2004	(EST) Estonia	106,42	77,92	36,02	0,46	1,00	32,81
2004	(FRA) France	80,30	58,15	28,30	0,49	1,00	69,36
2004	(HUN) Hungary	48,75	41,16	28,76	0,70	0,00	18,06
2004	(IRE) Ireland	42,17	6,52	2,98	0,46	1,00	51,45
2004	(ITA) Italy	15,24	11,59	5,50	0,47	1,00	13,11
2004	(LTU) Lithuania	31,66	28,10	12,71	0,45	1,00	10,61
2004	(LUX) Luxembourg	67,81	34,75	14,51	0,42	1,00	86,72
2004	(LVA) Latvia	58,16	42,29	18,51	0,44	1,00	21,80
2004	(NED) Netherlands	30,42	15,24	2,83	0,19	1,00	105,28
2004	(OST) Austria	30,67	16,05	9,40	0,59	1,00	24,83
2004	(POL) Poland	15,73	14,97	9,88	0,66	1,00	12,52
2004	(POR) Portugal	24,01	15,28	2,84	0,19	1,00	13,18
2004	(SUO) Finland	46,41	46,23	23,62	0,51	1,00	26,62
2004	(SVE) Sweden	87,67	70,50	29,97	0,43	1,00	54,72
2004	(SVN) Slovenia	10,84	3,10	1,51	0,49	1,00	12,09
2004	(UKI) United Kingdo	143,07	60,86	30,23	0,50	1,00	121,69

Density	sust-densi1	sust-densi2	sust-dentotatl	environsusten	Farm Net In	income-ind	inco-ind-equa
2,62	1,00	0,00	0,00	0,00	71029,00	56579,58	53154,79
0,49	0,00	1,00	0,00	0,00	43439,00	29267,52	29246,34
0,99	1,00	1,00	1,00	1,00	38436,00	32323,02	36900,90
0,61	1,00	1,00	1,00	1,00	9772,00	8530,89	9511,27
0,71	1,00	1,00	1,00	1,00	34995,00	29470,38	29534,09
0,26	0,00	1,00	0,00	0,00	8499,00	4651,42	8125,15
0,84	1,00	1,00	1,00	1,00	39359,00	33310,01	32069,69
0,40	0,00	1,00	0,00	0,00	22132,00	18417,29	18160,60
1,33	1,00	1,00	1,00	0,00	24839,00	21682,77	24501,05
0,91	1,00	1,00	1,00	1,00	37009,00	30224,04	28475,38
0,24	0,00	1,00	0,00	0,00	9514,00	5860,87	7291,06
1,45	1,00	1,00	1,00	1,00	58394,00	40088,63	39178,70
0,35	0,00	1,00	0,00	0,00	9776,00	5607,46	7419,44
3,70	1,00	0,00	0,00	0,00	82296,00	69624,87	73214,75
0,91	1,00	1,00	1,00	1,00	32339,00	25041,24	24580,94
0,61	1,00	1,00	1,00	0,00	8943,00	6415,90	6690,59
0,67	1,00	1,00	1,00	1,00	18584,00	14963,23	14405,39
0,45	0,00	1,00	0,00	0,00	21599,00	17094,52	15989,32
0,56	1,00	1,00	1,00	1,00	9229,00	7273,34	14956,49
1,03	1,00	1,00	1,00	1,00	10113,00	8069,27	6549,56
0,82	1,00	1,00	1,00	1,00	42474,00	32294,54	39619,28
2,75	1,00	0,00	0,00	0,00	61523,00	49730,00	51046,06
0,46	0,00	1,00	0,00	0,00	41845,00	29225,16	28607,51
1,01	1,00	1,00	1,00	1,00	48763,00	41478,79	36193,34
0,58	1,00	1,00	1,00	1,00	11909,00	10491,65	9517,55
0,68	1,00	1,00	1,00	1,00	34750,00	29597,81	29677,29
0,31	0,00	1,00	0,00	0,00	20508,00	11598,89	5047,68
0,84	1,00	1,00	1,00	1,00	36028,00	30829,38	28726,78
0,39	0,00	1,00	0,00	0,00	22055,00	17903,91	18009,54
1,35	1,00	1,00	1,00	0,00	31012,00	27319,34	23578,72
0,94	1,00	1,00	1,00	1,00	32298,00	26726,72	28063,77
0,26	0,00	1,00	0,00	0,00	13769,00	8721,26	7183,60
1,39	1,00	1,00	1,00	1,00	54495,00	38268,78	35054,03
0,32	0,00	1,00	0,00	0,00	15789,00	9231,41	7708,10
3,74	1,00	0,00	0,00	0,00	89784,00	76804,63	69225,39
0,93	1,00	1,00	1,00	1,00	30648,00	24120,64	22647,57
0,65	1,00	1,00	1,00	0,00	9629,00	6965,28	6402,38
0,62	1,00	1,00	1,00	1,00	16959,00	13847,55	13964,04
0,46	0,00	1,00	0,00	0,00	18526,00	14884,12	14116,50
0,65	1,00	1,00	1,00	1,00	26787,00	22639,64	15862,35
1,05	1,00	1,00	1,00	1,00	6213,00	5029,84	5685,96
0,89	1,00	1,00	1,00	1,00	46798,00	46944,01	36846,27
2,68	1,00	0,00	0,00	0,00	56954,00	46828,60	45336,07
0,45	0,00	1,00	0,00	0,00	37162,00	27329,85	28744,00
1,02	1,00	1,00	1,00	1,00	40462,00	34778,22	34520,74
0,59	1,00	1,00	1,00	1,00	10754,00	9530,11	9706,19
0,69	1,00	1,00	1,00	1,00	34702,00	29963,69	28854,41
0,30	0,00	1,00	0,00	0,00	-1889,00	-1107,27	4821,50

0,85	1,00	1,00	1,00	1,00	25640,00	22040,95	27727,95
0,37	0,00	1,00	0,00	0,00	20886,00	17707,40	16795,51
1,31	1,00	1,00	1,00	0,00	24396,00	21734,05	24348,64
0,91	1,00	1,00	1,00	1,00	32695,00	27240,56	26941,70
0,25	0,00	1,00	0,00	0,00	10553,00	6968,69	8031,62
1,36	1,00	1,00	1,00	1,00	37526,00	26804,69	34667,48
0,31	0,00	1,00	0,00	0,00	13761,00	8285,44	8557,21
3,80	1,00	0,00	0,00	0,00	70703,00	61246,67	64758,98
0,87	1,00	1,00	1,00	1,00	23595,00	18780,83	19769,96
0,64	1,00	1,00	1,00	0,00	7726,00	5825,96	6151,09
0,62	1,00	1,00	1,00	1,00	15782,00	13081,33	13544,14
0,49	0,00	1,00	0,00	0,00	12813,00	10370,85	13067,06
0,69	1,00	1,00	1,00	1,00	20832,00	17674,06	18444,64
1,07	1,00	1,00	1,00	1,00	4814,00	3958,76	4273,55
0,86	1,00	1,00	1,00	1,00	32105,00	31300,26	34578,53
2,52	1,00	0,00	0,00	0,00	47156,00	39449,61	43249,99
0,45	0,00	1,00	0,00	0,00	39847,00	29676,99	33286,88
1,01	1,00	1,00	1,00	1,00	31397,00	27305,22	32184,18
0,60	1,00	1,00	1,00	1,00	10290,00	9096,81	9618,59
0,66	1,00	1,00	1,00	1,00	31171,00	27001,73	24883,33
0,31	0,00	1,00	0,00	0,00	6653,00	3972,88	2313,04
0,84	1,00	1,00	1,00	1,00	35079,00	30313,53	27187,44
0,42	0,00	1,00	0,00	0,00	17346,00	14775,23	17006,22
1,19	1,00	1,00	1,00	0,00	27012,00	23992,53	23739,95
0,91	1,00	1,00	1,00	1,00	31875,00	26857,82	27430,25
0,26	0,00	1,00	0,00	0,00	12526,00	8404,93	7348,91
1,36	1,00	1,00	1,00	1,00	54080,00	38928,97	33649,92
0,34	0,00	1,00	0,00	0,00	13428,00	8154,79	7517,36
3,73	1,00	0,00	0,00	0,00	64613,00	56225,63	55276,05
0,85	1,00	1,00	1,00	1,00	20276,00	16408,40	17953,31
0,65	1,00	1,00	1,00	1,00	7808,00	5662,01	5950,01
0,58	1,00	1,00	1,00	1,00	16253,00	13703,53	12930,32
0,49	0,00	1,00	0,00	0,00	17198,00	13946,21	13579,36
0,70	1,00	1,00	1,00	1,00	17653,00	15020,23	13808,97
1,02	1,00	1,00	1,00	1,00	4625,00	3832,05	3864,24
0,86	1,00	1,00	1,00	1,00	27421,00	25491,33	30392,02
2,44	1,00	0,00	0,00	0,00	51276,00	43471,76	44340,52
0,44	0,00	1,00	0,00	0,00	56459,00	42853,81	35914,59
1,03	1,00	1,00	1,00	1,00	38968,00	34469,10	35961,76
0,60	1,00	1,00	1,00	1,00	11611,00	10228,85	9395,83
0,54	1,00	1,00	1,00	1,00	20305,00	17684,56	21340,61
0,32	0,00	1,00	0,00	0,00	6746,00	4073,49	6222,51
0,83	1,00	1,00	1,00	1,00	33420,00	29207,84	29092,71
0,38	0,00	1,00	0,00	0,00	21322,00	18536,02	16385,62
1,19	1,00	1,00	1,00	0,00	26627,00	25493,27	23396,88
0,88	1,00	1,00	1,00	1,00	33149,00	28192,36	24901,83
0,27	0,00	1,00	0,00	0,00	9936,00	6673,10	7941,24
1,35	1,00	1,00	1,00	1,00	48833,00	35216,10	36255,38
0,33	0,00	1,00	0,00	0,00	10064,00	6111,84	6743,84
3,74	1,00	0,00	0,00	0,00	55141,00	48355,84	55632,43

0,84	1,00	1,00	1,00	1,00	22552,00	18670,69	18756,12
0,66	1,00	1,00	1,00	0,00	8706,00	6362,06	6435,03
0,56	1,00	1,00	1,00	1,00	13957,00	12006,10	12560,94
0,52	1,00	1,00	1,00	1,00	19919,00	16421,03	14858,16
0,66	1,00	1,00	1,00	1,00	10327,00	8732,62	12361,55
1,01	1,00	1,00	1,00	1,00	4543,00	3801,92	3931,81
0,79	1,00	1,00	1,00	1,00	36772,00	34384,47	35024,46
2,50	1,00	0,00	0,00	0,00	58513,00	50100,18	49800,09
0,44	0,00	1,00	0,00	0,00	47882,00	35212,96	36874,84
1,02	1,00	1,00	1,00	1,00	51177,00	46110,97	42060,75
0,59	1,00	1,00	1,00	1,00	10247,00	8861,83	9553,06
0,57	1,00	1,00	1,00	1,00	22249,00	19335,54	18563,48
0,30	0,00	1,00	0,00	0,00	17086,00	10621,14	10683,58
0,82	1,00	1,00	1,00	1,00	31578,00	27756,76	33275,35
0,35	0,00	1,00	0,00	0,00	18293,00	15845,62	17065,75
1,17	1,00	1,00	1,00	1,00	21650,00	20704,85	22519,41
0,92	1,00	1,00	1,00	1,00	22900,00	19655,31	23011,91
0,26	0,00	1,00	0,00	0,00	12903,00	8745,68	8820,32
1,35	1,00	1,00	1,00	1,00	46730,00	34621,07	33431,36
0,30	0,00	1,00	0,00	0,00	9650,00	5964,88	6847,47
3,76	1,00	0,00	0,00	0,00	70878,00	62315,82	57312,71
0,75	1,00	1,00	1,00	1,00	25048,00	21189,26	21430,15
0,62	1,00	1,00	1,00	0,00	9867,00	7281,02	7217,20
0,54	1,00	1,00	1,00	1,00	13822,00	11973,18	11992,64
0,47	0,00	1,00	0,00	0,00	16956,00	14207,23	16378,61
0,68	1,00	1,00	1,00	1,00	16296,00	13331,79	11759,18
1,03	1,00	1,00	1,00	1,00	4950,00	4161,45	4081,13
0,76	1,00	1,00	1,00	1,00	43901,00	45197,58	42832,07
2,50	1,00	0,00	0,00	0,00	64381,00	55828,33	50654,68
0,44	0,00	1,00	0,00	0,00	45210,00	32557,74	34790,57
1,05	1,00	1,00	1,00	1,00	49635,00	45602,19	42397,73
0,62	1,00	1,00	1,00	1,00	11330,00	9568,50	9572,84
0,56	1,00	1,00	1,00	1,00	21399,00	18670,35	19043,70
0,29	0,00	1,00	0,00	0,00	26835,00	17356,09	14452,62
0,83	1,00	1,00	1,00	1,00	48386,00	42861,45	37643,29
0,34	0,00	1,00	0,00	0,00	19348,00	16815,62	17142,16
1,19	1,00	1,00	1,00	0,00	22064,00	21360,12	21926,79
0,90	1,00	1,00	1,00	1,00	24406,00	21188,07	20290,88
0,26	0,00	1,00	0,00	0,00	16075,00	11042,17	10288,60
1,30	1,00	1,00	1,00	1,00	40427,00	30456,91	34711,54
0,27	0,00	1,00	0,00	0,00	13475,00	8465,68	7625,82
3,69	1,00	0,00	0,00	0,00	68802,00	61266,47	53410,41
0,78	1,00	1,00	1,00	1,00	28419,00	24430,52	23990,70
0,62	1,00	1,00	1,00	0,00	10873,00	8008,52	7792,96
0,55	1,00	1,00	1,00	1,00	13546,00	11998,63	11679,37
0,47	0,00	1,00	0,00	0,00	21537,00	18507,55	17310,47
0,69	1,00	1,00	1,00	1,00	15899,00	13213,14	13765,89
1,02	1,00	1,00	1,00	1,00	5011,00	4280,04	4821,18
0,81	1,00	1,00	1,00	1,00	48564,00	48914,15	53825,83
2,66	1,00	0,00	0,00	0,00	52064,00	46035,52	53673,44

0,45	0,00	1,00	0,00	0,00	51112,00	36601,02	28794,24
1,02	1,00	1,00	1,00	1,00	38051,00	35480,04	38521,13
0,71	1,00	1,00	1,00	1,00	12228,00	10288,20	10534,42
0,67	1,00	1,00	1,00	1,00	21946,00	19125,22	19041,92
0,29	0,00	1,00	0,00	0,00	22860,00	15380,62	15259,87
0,84	1,00	1,00	1,00	1,00	47218,00	42311,67	41586,68
0,36	0,00	1,00	0,00	0,00	21671,00	18765,23	15694,85
1,11	1,00	1,00	1,00	1,00	23950,00	23715,41	20827,17
0,76	1,00	1,00	1,00	1,00	22719,00	20029,27	20597,21
0,28	0,00	1,00	0,00	0,00	15692,00	11077,95	11192,26
1,31	1,00	1,00	1,00	1,00	50548,00	39056,65	30374,47
0,28	0,00	1,00	0,00	0,00	12793,00	8446,88	8091,31
3,55	1,00	0,00	0,00	0,00	40570,00	36648,95	50744,14
0,81	1,00	1,00	1,00	1,00	30037,00	26352,34	23872,37
0,70	1,00	1,00	1,00	0,00	10887,00	8089,35	7838,21
0,58	1,00	1,00	1,00	1,00	12543,00	11066,30	11419,33
0,53	1,00	1,00	1,00	1,00	21717,00	19216,61	19860,26
0,63	1,00	1,00	1,00	1,00	16951,00	14752,74	14523,38
1,13	1,00	1,00	1,00	1,00	7017,00	6022,04	5451,55
0,83	1,00	1,00	1,00	1,00	61823,00	67365,76	58056,24
2,69	1,00	0,00	0,00	0,00	65713,00	59156,47	48230,45
0,45	0,00	1,00	0,00	0,00	23382,00	17223,95	20873,24
1,01	1,00	1,00	1,00	1,00	36587,00	34481,15	30665,97
0,79	1,00	1,00	1,00	1,00	13851,00	11746,55	11072,79
0,68	1,00	1,00	1,00	1,00	22186,00	19330,19	18703,50
0,30	0,00	1,00	0,00	0,00	18395,00	13042,88	12148,30
0,83	1,00	1,00	1,00	1,00	43761,00	39586,94	32859,71
0,36	0,00	1,00	0,00	0,00	13188,00	11503,69	12174,05
1,10	1,00	1,00	1,00	1,00	17319,00	17405,99	18916,07
0,74	1,00	1,00	1,00	1,00	22969,00	20574,30	20393,03
0,28	0,00	1,00	0,00	0,00	15401,00	11456,65	10828,02
1,29	1,00	1,00	1,00	1,00	26693,00	21609,85	27433,23
0,28	0,00	1,00	0,00	0,00	10478,00	7361,36	7056,05
3,62	1,00	0,00	0,00	0,00	60014,00	54317,00	37333,39
0,79	1,00	1,00	1,00	1,00	23320,00	20834,26	22458,56
0,71	1,00	1,00	1,00	0,00	9985,00	7416,77	6929,12
0,53	1,00	1,00	1,00	1,00	12721,00	11193,04	10766,14
0,52	1,00	1,00	1,00	1,00	24068,00	21856,63	18318,09
0,66	1,00	1,00	1,00	1,00	16770,00	15604,27	11986,49
1,10	1,00	1,00	1,00	1,00	6980,00	6052,56	6062,79
0,82	1,00	1,00	1,00	1,00	52641,00	57888,80	57304,47
2,61	1,00	0,00	0,00	0,00	43062,00	39499,37	46444,13
0,46	0,00	1,00	0,00	0,00	11607,00	8794,75	15648,62
1,04	1,00	1,00	1,00	1,00	23233,00	22036,71	27620,09
0,81	1,00	1,00	1,00	1,00	13098,00	11183,60	11614,01
0,64	1,00	1,00	1,00	1,00	20233,00	17655,09	19749,78
0,31	0,00	1,00	0,00	0,00	11110,00	8021,40	11128,85
0,84	1,00	1,00	1,00	1,00	18244,00	16680,52	29330,45
0,36	0,00	1,00	0,00	0,00	6878,00	6253,23	10581,96
1,12	1,00	1,00	1,00	1,00	16053,00	15626,82	16829,32

0,83	1,00	1,00	1,00	1,00	22870,00	20575,52	20332,36
0,29	0,00	1,00	0,00	0,00	13078,00	9949,45	10926,79
1,26	1,00	1,00	1,00	1,00	25790,00	21633,20	26374,03
0,32	0,00	1,00	0,00	0,00	7660,00	5359,91	6759,78
3,65	1,00	0,00	0,00	0,00	23023,00	21034,22	34343,29
0,84	1,00	1,00	1,00	1,00	22402,00	20189,07	23079,08
0,72	1,00	1,00	1,00	0,00	6445,00	5281,23	6121,55
0,53	1,00	1,00	1,00	1,00	11337,00	10039,09	10462,66
0,52	1,00	1,00	1,00	1,00	15236,00	13881,04	18102,38
0,66	1,00	1,00	1,00	1,00	5362,00	5602,44	15517,49
1,14	1,00	1,00	1,00	1,00	7124,00	6113,77	5713,02
0,82	1,00	1,00	1,00	1,00	40424,00	46658,83	51177,12
2,54	1,00	0,00	0,00	0,00	44107,00	40676,55	44819,33
0,53	1,00	1,00	1,00	1,00	28426,00	20927,14	20028,18
1,07	1,00	1,00	1,00	1,00	27271,00	26342,39	30269,96
0,83	1,00	1,00	1,00	1,00	13601,00	11911,87	12184,27
0,64	1,00	1,00	1,00	1,00	25479,00	22264,07	21824,26
0,31	0,00	1,00	0,00	0,00	17099,00	12322,27	12649,00
0,87	1,00	1,00	1,00	1,00	34676,00	31723,88	29668,36
0,36	0,00	1,00	0,00	0,00	16444,00	13988,95	10609,49
1,13	1,00	1,00	1,00	1,00	18788,00	17455,16	17636,47
0,85	1,00	1,00	1,00	1,00	21695,00	19847,25	21264,24
0,35	0,00	1,00	0,00	0,00	15459,00	11374,26	11434,00
1,31	1,00	1,00	1,00	1,00	42186,00	35879,02	33979,71
0,35	0,00	1,00	0,00	0,00	12137,00	7558,08	7848,97
3,53	1,00	0,00	0,00	0,00	30230,00	27678,66	30481,74
0,83	1,00	1,00	1,00	1,00	30723,00	28213,90	25499,90
0,68	1,00	1,00	1,00	0,00	8197,00	5666,65	6220,92
0,57	1,00	1,00	1,00	1,00	11345,00	10155,85	9863,02
0,54	1,00	1,00	1,00	1,00	20026,00	18569,48	19093,75
0,69	1,00	1,00	1,00	1,00	26163,00	25345,76	18238,71
1,17	1,00	1,00	1,00	1,00	5604,00	4972,71	5919,15
0,83	1,00	1,00	1,00	1,00	45090,00	48983,74	47892,05
2,60	1,00	0,00	0,00	0,00	57763,00	54282,07	48870,63
0,52	1,00	1,00	1,00	1,00	36342,00	30362,64	24098,51
1,06	1,00	1,00	1,00	1,00	43529,00	42430,77	33730,18
0,88	1,00	1,00	1,00	1,00	14726,00	13457,32	12887,69
0,59	1,00	1,00	1,00	1,00	28601,00	25553,62	23445,75
0,31	0,00	1,00	0,00	0,00	22847,00	17603,34	13387,39
0,86	1,00	1,00	1,00	1,00	43354,00	40600,67	34888,10
0,38	0,00	1,00	0,00	0,00	13018,00	11586,29	10946,57
1,14	1,00	1,00	1,00	1,00	21411,00	19827,44	18038,73
0,73	1,00	1,00	1,00	1,00	24948,00	23369,94	21275,92
0,36	0,00	1,00	0,00	0,00	16078,00	12978,30	10456,57
1,24	1,00	1,00	1,00	1,00	50284,00	44426,90	38436,45
0,35	0,00	1,00	0,00	0,00	15278,00	10628,92	9594,18
3,51	1,00	0,00	0,00	0,00	45620,00	42732,35	38657,92
0,80	1,00	1,00	1,00	1,00	30007,00	28096,75	26701,81
0,72	1,00	1,00	1,00	0,00	9979,00	7714,87	6509,59
0,61	1,00	1,00	1,00	1,00	10315,00	9394,12	9643,51

0,55	1,00	1,00	1,00	1,00	25990,00	24830,74	20359,45
0,64	1,00	1,00	1,00	1,00	24703,00	23767,92	19184,61
1,17	1,00	1,00	1,00	1,00	7197,00	6670,97	4914,16
0,83	1,00	1,00	1,00	1,00	50562,00	48033,58	43235,88
2,55	1,00	0,00	0,00	0,00	53925,00	51653,26	50941,84
0,54	1,00	1,00	1,00	1,00	23860,00	21005,75	22705,06
1,04	1,00	1,00	1,00	1,00	32681,00	32417,39	34752,62
0,82	1,00	1,00	1,00	1,00	14065,00	13293,87	13506,60
0,56	1,00	1,00	1,00	1,00	24374,00	22519,57	22597,14
0,31	0,00	1,00	0,00	0,00	11819,00	10236,55	14008,98
0,86	1,00	1,00	1,00	1,00	33672,00	32339,76	33966,80
0,38	0,00	1,00	0,00	0,00	7362,00	7264,46	8145,63
1,15	1,00	1,00	1,00	1,00	17960,00	16833,59	18116,67
0,78	1,00	1,00	1,00	1,00	21470,00	20610,56	21489,37
0,39	0,00	1,00	0,00	0,00	8007,00	7017,16	8912,50
1,20	1,00	1,00	1,00	1,00	39035,00	35003,42	38443,77
0,36	0,00	1,00	0,00	0,00	12678,00	10595,53	10273,52
3,37	1,00	0,00	0,00	0,00	47655,00	45562,74	42295,51
0,79	1,00	1,00	1,00	1,00	24860,00	23794,78	24917,69
0,74	1,00	1,00	1,00	0,00	7450,00	6147,25	6303,36
0,53	1,00	1,00	1,00	1,00	10003,00	9380,55	8952,82
0,53	1,00	1,00	1,00	1,00	18007,00	17678,12	20742,25
0,68	1,00	1,00	1,00	1,00	8526,00	8440,17	14727,00
1,17	1,00	1,00	1,00	1,00	3209,00	3098,81	4898,19
0,83	1,00	1,00	1,00	1,00	34516,00	32690,31	37564,83
2,61	1,00	0,00	0,00	0,00	47865,00	46890,19	48135,82
0,55	1,00	1,00	1,00	1,00	17940,00	16746,81	20685,18
1,02	1,00	1,00	1,00	1,00	29530,00	29409,69	30598,03
0,77	1,00	1,00	1,00	1,00	14076,00	13768,61	13090,49
0,57	1,00	1,00	1,00	1,00	20526,00	19718,25	22635,27
0,32	0,00	1,00	0,00	0,00	15026,00	14187,04	13381,86
0,86	1,00	1,00	1,00	1,00	29518,00	28959,96	30437,91
0,43	0,00	1,00	0,00	0,00	5818,00	5586,14	6425,20
1,24	1,00	1,00	1,00	1,00	18241,00	17688,96	17372,52
0,81	1,00	1,00	1,00	1,00	20900,00	20487,62	20354,73
0,33	0,00	1,00	0,00	0,00	7207,00	6742,03	6948,73
1,23	1,00	1,00	1,00	1,00	37405,00	35900,98	35929,13
0,37	0,00	1,00	0,00	0,00	10212,00	9596,11	9704,88
3,45	1,00	0,00	0,00	0,00	39358,00	38591,43	38067,72
0,79	1,00	1,00	1,00	1,00	23441,00	22861,55	23349,44
0,73	1,00	1,00	1,00	0,00	5834,00	5047,97	5768,41
0,56	1,00	1,00	1,00	1,00	8354,00	8083,80	8550,45
0,56	1,00	1,00	1,00	1,00	19901,00	19717,89	19269,00
0,63	1,00	1,00	1,00	1,00	11848,00	11972,91	8540,36
1,15	1,00	1,00	1,00	1,00	4989,00	4924,80	4694,20
0,84	1,00	1,00	1,00	1,00	32672,00	31970,59	30803,30
2,60	1,00	0,00	0,00	0,00	45864,00	45864,00	46377,09
0,58	1,00	1,00	1,00	1,00	24303,00	24303,00	20524,90
1,07	1,00	1,00	1,00	1,00	29967,00	29967,00	29688,35
0,80	1,00	1,00	1,00	1,00	12209,00	12209,00	12988,80

0,60	1,00	1,00	1,00	1,00	25668,00	25668,00	22693,12
0,31	0,00	1,00	0,00	0,00	15722,00	15722,00	14954,52
0,86	1,00	1,00	1,00	1,00	30014,00	30014,00	29486,98
0,37	0,00	1,00	0,00	0,00	6425,00	6425,00	6005,57
1,22	1,00	1,00	1,00	1,00	17595,00	17595,00	17641,98
0,86	1,00	1,00	1,00	1,00	19966,00	19966,00	20226,81
0,34	0,00	1,00	0,00	0,00	7087,00	7087,00	6914,52
1,28	1,00	1,00	1,00	1,00	36883,00	36883,00	36391,99
0,37	0,00	1,00	0,00	0,00	8923,00	8923,00	9259,55
3,46	1,00	0,00	0,00	0,00	30049,00	30049,00	34320,21
0,81	1,00	1,00	1,00	1,00	23392,00	23392,00	23126,78
0,80	1,00	1,00	1,00	1,00	6110,00	6110,00	5578,99
0,55	1,00	1,00	1,00	1,00	8187,00	8187,00	8135,40
0,57	1,00	1,00	1,00	1,00	20411,00	20411,00	20064,45
0,62	1,00	1,00	1,00	1,00	5208,00	5208,00	8590,45
1,12	1,00	1,00	1,00	1,00	6059,00	6059,00	5491,90
0,85	1,00	1,00	1,00	1,00	27749,00	27749,00	29859,79

Total assets (S	totassets-ind	totasseindequali	Total fixed assets (	sharefixassets	Land, permane	Total liabilities
963854,00	768033,02	764379,59	793267,00	0,82	462041,00	221372,00
747346,00	503595,54	513301,49	509000,00	0,68	169954,00	219993,00
966056,00	811979,37	809122,49	847096,00	0,88	567516,00	226536,00
112574,00	98277,31	97933,80	106023,00	0,94	73713,00	49,00
370015,00	311463,42	310905,87	232927,00	0,63	182242,00	12059,00
350516,00	192145,59	184944,22	261675,00	0,75	77895,00	132728,00
450417,00	381114,51	375777,73	258024,00	0,57	65114,00	188499,00
206566,00	171890,70	169770,55	131508,00	0,64	56344,00	31069,00
1013731,00	884965,49	879751,69	888275,00	0,88	778239,00	26077,00
508683,00	415390,76	418054,18	301859,00	0,59	247784,00	5848,00
134080,00	82611,45	81878,27	92182,00	0,69	38818,00	26192,00
1239375,00	850762,86	838153,27	1078424,00	0,87	562739,00	293089,00
183227,00	105095,50	96914,06	137849,00	0,75	50229,00	62885,00
3123033,00	2642303,90	2370978,55	2784609,00	0,89	2248962,00	878811,00
580868,00	450000,67	420653,11	458860,00	0,79	181656,00	85920,00
187180,00	134304,65	131599,93	167299,00	0,89	99954,00	10257,00
101917,00	82067,75	82594,38	84330,00	0,83	48271,00	3743,00
519812,00	411475,84	397887,15	426640,00	0,82	246935,00	143042,00
1131414,00	891731,69	954167,38	962009,00	0,85	632334,00	261528,00
226539,00	180734,52	182156,59	205449,00	0,91	111473,00	11353,00
1979137,00	1504433,18	1710198,83	1795266,00	0,91	1548800,00	215727,00
940830,00	760726,15	756024,34	766110,00	0,81	463806,00	192835,00
748762,00	523007,43	513079,45	506721,00	0,68	164212,00	232230,00
948330,00	806265,61	803478,70	830219,00	0,88	560519,00	212679,00
110773,00	97590,30	101524,39	104184,00	0,94	70541,00	12,00
364523,00	310348,32	308247,14	247379,00	0,68	194893,00	11054,00
313778,00	177742,86	180380,03	233258,00	0,74	66658,00	109227,00
432991,00	370440,95	376508,71	255834,00	0,59	69573,00	184494,00
206526,00	167650,40	170079,72	130643,00	0,63	52438,00	30820,00
992697,00	874537,90	887859,23	874643,00	0,88	769501,00	23925,00
508457,00	420717,60	411887,02	295971,00	0,58	245198,00	4356,00
128089,00	81145,09	81773,14	85058,00	0,66	33915,00	22146,00
1175701,00	825543,68	831037,62	1027279,00	0,87	525828,00	270241,00
151768,00	88732,62	94564,89	113050,00	0,74	43364,00	47819,00
2454369,00	2099653,20	2264395,24	2124146,00	0,87	1644398,00	779369,00
496975,00	391305,55	399410,63	377030,00	0,76	105124,00	62890,00
178166,00	128895,21	129710,30	158693,00	0,89	93371,00	9522,00
101789,00	83121,01	84301,13	84585,00	0,83	50152,00	3005,00
478252,00	384298,47	395382,28	393921,00	0,82	227986,00	134847,00
1202748,00	1016603,08	930142,93	1025769,00	0,85	658359,00	307206,00
226789,00	183578,65	178409,89	195904,00	0,86	110085,00	8327,00
1910458,00	1915964,47	1783001,53	1733034,00	0,91	1488501,00	214479,00
898902,00	739313,84	705613,99	727147,00	0,81	426600,00	182110,00
696994,00	512635,37	511494,79	467264,00	0,67	144555,00	214508,00
922114,00	792191,13	796311,51	811506,00	0,88	545276,00	211861,00
122653,00	108705,56	104992,26	116626,00	0,95	82517,00	41,00
350956,00	302929,68	302494,25	238998,00	0,68	185578,00	10813,00
291698,00	171251,63	174200,29	219869,00	0,75	63074,00	104498,00

439761,00	377970,66	375793,00	258873,00	0,59	69145,00	188087,00
201334,00	170698,07	165040,98	125951,00	0,63	50853,00	30343,00
1014731,00	904074,31	889772,06	893453,00	0,88	788530,00	23424,00
479576,00	399552,69	407086,06	298914,00	0,62	246863,00	4487,00
123496,00	81562,88	79642,29	81867,00	0,66	31182,00	23437,00
1143647,00	816806,31	833922,40	1005107,00	0,88	498879,00	275311,00
149256,00	89866,56	89848,44	112093,00	0,75	42460,00	47637,00
2367913,00	2051228,62	2075193,68	2058567,00	0,87	1595470,00	798869,00
448235,00	356925,68	368376,90	345024,00	0,77	100070,00	54320,00
166982,00	125931,02	126027,28	149168,00	0,89	87151,00	9153,00
105812,00	87714,62	86432,95	88753,00	0,84	54707,00	2735,00
482211,00	390372,53	390566,66	401489,00	0,83	226443,00	142124,00
1039633,00	882094,04	927080,11	866936,00	0,83	506545,00	311093,00
207851,00	170916,50	177375,15	189320,00	0,91	106625,00	6218,00
1978748,00	1928606,93	1909861,20	1802181,00	0,91	1555184,00	216347,00
737113,00	616801,98	654612,47	630469,00	0,86	330149,00	203136,00
669806,00	498841,58	494934,92	445299,00	0,66	126242,00	202034,00
909388,00	790477,79	792394,24	804851,00	0,89	538424,00	205088,00
122932,00	108680,94	110653,73	117346,00	0,95	81543,00	51,00
339756,00	294204,74	278123,21	243055,00	0,72	189289,00	10280,00
290736,00	173606,37	173799,54	214340,00	0,74	60034,00	99751,00
438601,00	378967,39	377130,47	261295,00	0,60	69051,00	186430,00
184062,00	156774,48	161024,29	118537,00	0,64	49439,00	30763,00
1002731,00	890703,97	917604,75	885214,00	0,88	779500,00	22510,00
475893,00	400987,89	403702,42	297742,00	0,63	248761,00	5010,00
113579,00	76218,89	79550,04	72817,00	0,64	29382,00	18215,00
1194111,00	859417,21	848602,64	1047161,00	0,88	506585,00	291252,00
149775,00	90946,13	88038,59	112085,00	0,75	40345,00	48480,00
2384353,00	2074699,21	2070664,31	2010897,00	0,84	1532115,00	818044,00
440883,00	356899,46	357055,12	342205,00	0,78	96664,00	54299,00
169941,00	123255,59	124046,97	152594,00	0,90	88053,00	9745,00
104920,00	88463,22	88774,26	88244,00	0,84	54016,00	3327,00
489483,00	397028,99	392602,28	404096,00	0,83	225616,00	134953,00
1037127,00	882543,21	873170,18	864505,00	0,83	492770,00	317851,00
214399,00	177630,29	175602,79	197564,00	0,92	110618,00	7213,00
2028317,00	1885012,19	1877513,92	1868717,00	0,92	1610586,00	213146,00
716722,00	607721,57	616293,33	621262,00	0,87	325587,00	192707,00
623691,00	473327,82	530878,14	411208,00	0,66	105085,00	187640,00
898461,00	794513,80	801898,80	790575,00	0,88	525757,00	192771,00
130049,00	114574,69	105843,66	124886,00	0,96	88360,00	114,00
272487,00	237235,21	253681,44	202467,00	0,74	159992,00	8173,00
292378,00	176540,63	175659,74	219348,00	0,75	57078,00	100739,00
428530,00	374453,34	381386,50	260576,00	0,61	67627,00	177170,00
178992,00	155600,31	156377,14	114742,00	0,64	47232,00	30123,00
1000614,00	958035,97	907097,28	881971,00	0,88	776636,00	22825,00
482789,00	410566,67	392004,83	312319,00	0,65	259844,00	5060,00
120403,00	80868,36	78011,39	81158,00	0,67	32052,00	18946,00
1206000,00	869584,39	875146,40	1059200,00	0,88	507566,00	306680,00
137188,00	83303,07	88883,13	100676,00	0,73	34440,00	44128,00
2378846,00	2086065,09	2097293,92	2001128,00	0,84	1523021,00	817435,00

431520,00	357340,21	366158,31	334472,00	0,78	92744,00	52763,00
168234,00	122954,28	122640,09	151017,00	0,90	85880,00	9622,00
104796,00	90144,94	92413,51	88840,00	0,85	52967,00	3208,00
473465,00	390405,31	386219,71	396696,00	0,84	217101,00	126816,00
1010858,00	854873,30	828170,94	856570,00	0,85	483429,00	314891,00
213015,00	178261,59	171340,49	196199,00	0,92	106230,00	6271,00
1945923,00	1818922,65	1834132,21	1791215,00	0,92	1538094,00	199631,00
729126,00	624356,42	600267,46	640027,00	0,88	327967,00	191880,00
843820,00	620465,01	553905,12	634477,00	0,75	111287,00	187722,00
911109,00	820704,81	802433,04	766850,00	0,84	505097,00	188087,00
109005,00	94275,36	100539,27	104219,00	0,96	68293,00	355,00
264312,00	229604,37	233820,77	201454,00	0,76	155942,00	6815,00
284481,00	176832,22	172377,40	206896,00	0,73	50523,00	93606,00
444631,00	390738,76	386347,11	261380,00	0,59	67441,00	176001,00
180962,00	156756,62	154764,62	111327,00	0,62	44853,00	30215,00
912340,00	872551,91	896875,16	853881,00	0,94	732049,00	23264,00
424692,00	364459,93	378220,09	306301,00	0,72	238147,00	3228,00
113525,00	76946,94	77958,92	74139,00	0,65	23921,00	17396,00
1210108,00	896437,61	886359,11	1025658,00	0,85	492094,00	289538,00
149503,00	92400,19	87537,28	100642,00	0,67	35410,00	46956,00
2424071,00	2131117,46	2112241,71	2104485,00	0,87	1571771,00	822443,00
454113,00	384235,24	374446,65	354824,00	0,78	94099,00	51412,00
164912,00	121710,39	119342,04	145525,00	0,88	81644,00	9917,00
113855,00	98632,37	94717,41	86759,00	0,76	52134,00	3034,00
443009,00	371224,83	377412,23	366572,00	0,83	202669,00	118120,00
913082,00	747096,30	792813,19	714926,00	0,78	381605,00	321448,00
188106,00	158129,58	165247,22	175550,00	0,93	93333,00	3752,00
1747485,00	1798461,80	1764129,73	1582844,00	0,91	1345130,00	166204,00
655798,00	568724,38	579826,54	569791,00	0,87	274390,00	187244,00
788751,00	567922,54	608008,54	589688,00	0,75	86363,00	189693,00
862291,00	792080,52	787926,77	723255,00	0,84	479079,00	173836,00
109847,00	92767,77	94357,30	105187,00	0,96	68626,00	398,00
268998,00	234622,73	244725,94	207980,00	0,77	159511,00	8007,00
253223,00	163759,35	163035,13	183587,00	0,73	45734,00	74366,00
444721,00	393849,23	388576,51	256619,00	0,58	66283,00	172791,00
174813,00	151936,93	152463,32	108102,00	0,62	45576,00	29325,00
888354,00	860037,59	834572,33	832893,00	0,94	710477,00	23134,00
414339,00	359633,67	354413,00	308377,00	0,74	238716,00	3045,00
110721,00	76061,47	77192,45	72990,00	0,66	21281,00	16302,00
1185433,00	893055,32	877504,85	997124,00	0,84	491267,00	267976,00
138347,00	86908,59	86455,54	89488,00	0,65	29415,00	44180,00
2380444,00	2119542,59	2077114,70	2068068,00	0,87	1512107,00	860029,00
443999,00	381764,49	376724,31	347220,00	0,78	89753,00	49221,00
153893,00	113361,44	115494,89	134781,00	0,88	76570,00	9854,00
107666,00	95374,93	94915,44	91390,00	0,85	55496,00	3759,00
431248,00	370606,53	368659,79	355737,00	0,82	192587,00	115020,00
934151,00	776469,97	751002,11	742280,00	0,79	404775,00	303091,00
186580,00	159350,49	159984,15	173070,00	0,93	89861,00	4730,00
1663482,00	1675004,74	1702210,20	1490889,00	0,90	1257417,00	160392,00
617896,00	546398,80	547483,00	534793,00	0,87	251371,00	185352,00

887709,00	635638,06	593089,35	659447,00	0,74	88655,00	207324,00
805491,00	750994,98	765009,09	682576,00	0,85	457938,00	159125,00
114131,00	96028,77	88793,73	109416,00	0,96	73277,00	562,00
309857,00	269950,72	263110,22	206561,00	0,67	167829,00	7669,00
220775,00	148513,83	149597,76	163896,00	0,74	40418,00	64610,00
425420,00	381141,56	382262,90	247993,00	0,58	67046,00	166239,00
171726,00	148696,41	143803,61	105013,00	0,61	41259,00	34738,00
778714,00	771127,49	812120,82	733510,00	0,94	626629,00	18372,00
384776,00	339145,39	336330,68	292371,00	0,76	226226,00	2871,00
111284,00	78568,94	78651,21	76240,00	0,69	20493,00	14818,00
1091214,00	843021,61	863916,58	906782,00	0,83	464019,00	224360,00
121256,00	80057,83	80830,72	79810,00	0,66	27064,00	38518,00
2192649,00	1980684,05	2042969,79	1894708,00	0,86	1382717,00	803023,00
414982,00	364173,21	366545,49	320748,00	0,77	82716,00	46448,00
149939,00	111412,83	111082,83	131551,00	0,88	73952,00	9044,00
102838,00	90739,02	91109,25	83349,00	0,81	51802,00	3989,00
411468,00	364148,01	365349,42	338583,00	0,82	171698,00	113828,00
837958,00	729440,06	723639,31	670534,00	0,80	376581,00	258998,00
189330,00	162472,37	165362,97	175566,00	0,93	92138,00	3350,00
1499014,00	1633164,08	1617055,19	1338034,00	0,89	1114451,00	156900,00
585714,00	527325,82	540127,02	505572,00	0,86	234069,00	178474,00
781547,00	575707,46	590541,23	587209,00	0,75	77730,00	180065,00
797912,00	751951,78	748171,24	677566,00	0,85	460042,00	155185,00
91480,00	77584,63	84292,13	86738,00	0,95	60618,00	566,00
326912,00	284757,21	281250,26	217663,00	0,67	176573,00	7863,00
192567,00	136520,11	149446,54	146513,00	0,76	35666,00	59102,00
411073,00	371797,92	371407,73	243027,00	0,59	66890,00	162926,00
149935,00	130777,48	133999,45	95949,00	0,64	37919,00	30699,00
801095,00	805197,39	790094,73	759717,00	0,95	651820,00	20336,00
346370,00	310212,98	313963,33	291123,00	0,84	217750,00	2670,00
109306,00	81323,22	78652,39	74244,00	0,68	19113,00	14195,00
1057038,00	855672,79	851298,36	876822,00	0,83	440532,00	209258,00
107514,00	75525,74	77981,86	69868,00	0,65	25417,00	32729,00
2241514,00	2028682,74	1951880,48	1941319,00	0,87	1454241,00	792626,00
395791,00	353698,78	368947,55	311216,00	0,79	84684,00	40447,00
146017,00	108474,22	109938,11	129431,00	0,89	72983,00	9023,00
99106,00	87213,80	87823,60	78997,00	0,80	49137,00	2992,00
397789,00	361293,71	354112,38	329125,00	0,83	164382,00	114092,00
714624,00	665007,92	669560,45	558008,00	0,78	300677,00	225413,00
200989,00	174266,06	166546,23	188671,00	0,94	100169,00	3847,00
1403393,00	1542996,75	1545211,96	1247934,00	0,89	1038544,00	139122,00
595945,00	546656,42	532347,57	517145,00	0,87	248863,00	171321,00
739401,00	560278,18	567137,92	555356,00	0,75	73873,00	169173,00
781844,00	741566,97	734105,50	666008,00	0,85	451907,00	149337,00
92832,00	79262,98	77962,47	88225,00	0,95	61629,00	729,00
331331,00	289042,84	290653,95	227812,00	0,69	186572,00	7939,00
226229,00	163305,68	146761,33	181865,00	0,80	40052,00	69138,00
395246,00	361283,71	365657,43	238852,00	0,60	64916,00	159299,00
134772,00	122524,46	130733,75	87304,00	0,65	32820,00	30714,00
815600,00	793959,29	824604,00	775102,00	0,95	669978,00	22485,00

325217,00	292531,62	293133,38	288262,00	0,89	217047,00	4037,00
99974,00	76065,01	76155,09	68755,00	0,69	17263,00	14537,00
1019581,00	855200,68	851322,28	841034,00	0,82	443240,00	176424,00
112004,00	78362,00	75285,56	74901,00	0,67	25349,00	38004,00
2020928,00	1846274,65	1818459,78	1775577,00	0,88	1313539,00	752078,00
431506,00	388970,65	373277,48	343617,00	0,80	98257,00	42744,00
134130,00	109927,28	107722,52	120072,00	0,90	66598,00	9236,00
96562,00	85517,96	85514,03	77474,00	0,80	48211,00	2774,00
369750,00	336895,43	346677,24	310024,00	0,84	149722,00	106971,00
587793,00	614233,36	624090,51	451964,00	0,77	247432,00	182336,00
189832,00	162900,27	180448,81	177745,00	0,94	92146,00	3401,00
1264592,00	1459475,04	1432667,58	1128415,00	0,89	939693,00	117683,00
567223,00	523060,46	524494,34	493715,00	0,87	259692,00	146467,00
768040,00	565428,14	568052,28	565072,00	0,74	67389,00	179460,00
733770,00	708797,76	720631,25	626846,00	0,85	430010,00	133862,00
87970,00	77039,79	78945,25	83656,00	0,95	58745,00	517,00
341294,00	298161,80	290096,75	218061,00	0,64	178090,00	7476,00
194911,00	140458,20	144845,55	156012,00	0,80	38489,00	59646,00
397814,00	363890,68	360280,34	239156,00	0,60	65537,00	148939,00
163289,00	138899,31	129686,98	99028,00	0,61	35618,00	43902,00
941425,00	874655,32	872993,53	898110,00	0,95	789726,00	24710,00
302483,00	276655,53	290090,57	268524,00	0,89	197511,00	4428,00
96602,00	71077,04	73210,52	63742,00	0,66	16324,00	14535,00
991281,00	843093,37	849857,77	816186,00	0,82	426419,00	181200,00
115589,00	71968,95	73238,70	74417,00	0,64	22389,00	42228,00
1726105,00	1580421,94	1656859,49	1494791,00	0,87	1078586,00	647592,00
410625,00	377163,00	382029,04	316776,00	0,77	99230,00	42005,00
151546,47	104766,06	106040,81	133519,47	0,88	67943,47	11551,00
93603,00	83810,32	85585,28	80516,00	0,86	50385,00	4199,00
368622,00	341842,58	339349,26	305551,00	0,83	142493,00	99037,00
612165,00	593030,26	590494,79	458036,00	0,75	249098,00	168575,00
230114,00	204180,12	177224,81	218626,00	0,95	96247,00	3253,00
1192532,00	1295530,95	1328493,00	1056531,00	0,89	895149,00	120500,00
536077,00	503766,13	488446,50	462326,00	0,86	243614,00	141198,00
692339,00	578450,53	590156,42	508682,00	0,73	54611,00	155583,00
729987,00	711529,02	706905,33	625897,00	0,86	434827,00	128557,00
88130,00	80532,97	79795,78	83698,00	0,95	60419,00	385,00
316880,00	283085,62	279141,67	194863,00	0,61	157224,00	7157,00
169748,00	130772,76	128068,74	130030,00	0,77	32695,00	47458,00
379840,00	355666,64	352881,16	225148,00	0,59	56554,00	141278,00
143419,00	127637,18	124640,32	89987,00	0,63	32756,00	41263,00
1026134,00	950365,97	903686,21	986877,00	0,96	891629,00	20406,00
321485,00	301084,57	296493,19	285674,00	0,89	219838,00	3663,00
89807,00	72489,52	64105,83	59668,00	0,66	17582,00	14412,00
963521,00	851279,25	836481,25	794763,00	0,82	421629,00	167081,00
99738,00	69385,14	69548,85	63539,00	0,64	20319,00	32448,00
1648284,00	1543881,87	1523287,91	1423482,00	0,86	1041836,00	613629,00
405737,00	379953,46	373306,44	315041,00	0,78	107743,00	37623,00
133776,10	103429,09	102327,37	118314,10	0,88	62630,10	9644,00
95977,00	87427,55	85284,38	80517,00	0,84	48342,00	3650,00

355115,00	339309,77	329611,79	290931,00	0,82	133311,00	93106,00
586439,00	564220,76	576830,30	432274,00	0,74	243140,00	177689,00
177564,00	164594,04	177762,39	166699,00	0,94	93813,00	3541,00
1295249,00	1230473,01	1177088,49	1143431,00	0,88	944416,00	133142,00
457783,00	438512,92	455311,14	389968,00	0,85	196461,00	127802,00
711669,00	626590,58	601249,40	542442,00	0,76	52485,00	157216,00
706066,00	700389,19	702314,14	607283,00	0,86	420246,00	124019,00
86564,00	81814,59	80646,96	81907,00	0,95	58619,00	393,00
277282,00	256177,60	252967,52	166211,00	0,60	129935,00	6644,00
130449,00	112975,27	121873,79	103063,00	0,79	25731,00	37035,00
353108,00	339086,14	343223,77	212806,00	0,60	55549,00	133307,00
108826,00	107384,46	117447,44	71380,00	0,66	27611,00	27463,00
945225,00	886037,34	851045,80	908431,00	0,96	826195,00	18677,00
324805,00	311739,47	309647,56	286899,00	0,88	219339,00	3762,00
55628,00	48750,94	55541,82	40343,00	0,73	13178,00	6037,00
908950,00	815071,14	838137,58	750568,00	0,83	407883,00	156588,00
80524,00	67292,46	67936,97	51502,00	0,64	17487,00	24666,00
1511920,00	1445559,93	1491620,36	1316114,00	0,87	984186,00	535286,00
379005,00	362802,85	365608,03	287791,00	0,76	88549,00	34081,00
119720,56	98786,96	100502,77	107009,56	0,89	57474,56	7673,00
90214,00	84615,26	83769,40	75699,00	0,84	45675,00	3261,00
313388,00	307683,02	311891,63	260087,00	0,83	124816,00	82021,00
579048,00	573239,88	556807,36	441711,00	0,76	215463,00	164097,00
170358,00	164513,00	169128,48	163806,00	0,96	92876,00	3106,00
1061437,00	1005261,52	1062541,56	923040,00	0,87	750020,00	121368,00
432476,00	423654,37	423410,43	366058,00	0,85	183691,00	119445,00
641357,00	598707,09	600603,56	479609,00	0,75	43281,00	147760,00
697903,00	695024,20	697828,80	604829,00	0,87	422761,00	116872,00
81373,00	79593,30	79902,30	76774,00	0,94	55928,00	367,00
228643,00	219639,32	231146,64	154593,00	0,68	118348,00	5957,00
129078,00	121873,34	118277,54	98150,00	0,76	23413,00	33528,00
341408,00	334918,51	337556,55	206536,00	0,60	54134,00	131896,00
122194,00	117320,68	115616,05	81094,00	0,66	27949,00	34489,00
739080,00	716734,11	720635,81	704054,00	0,95	628461,00	15462,00
322485,00	316118,63	304062,70	286051,00	0,89	216938,00	3531,00
48517,00	45385,00	45848,98	34303,00	0,71	12707,00	3817,00
883635,00	848062,35	834793,50	730733,00	0,83	392247,00	149469,00
71438,00	67133,30	65040,58	45662,00	0,64	14989,00	20263,00
1514835,00	1485419,28	1456494,40	1344659,00	0,89	1031812,00	507303,00
363016,00	354067,77	356076,21	283521,00	0,78	81004,00	35696,00
114762,65	99292,27	100343,76	103687,65	0,90	55006,65	7219,00
81907,00	79265,38	82840,55	69371,00	0,85	39393,00	2547,00
291353,00	288682,10	289370,04	238693,00	0,82	111656,00	78546,00
527412,00	532961,45	516253,77	447817,00	0,85	232060,00	149202,00
180607,00	178278,40	175412,80	173737,00	0,96	98790,00	3659,00
972826,00	951890,14	940259,22	836313,00	0,86	675868,00	125504,00
408064,00	408064,00	415859,19	341863,00	0,84	163342,00	120272,00
576513,00	576513,00	587610,04	432358,00	0,75	41549,00	137624,00
698073,00	698073,00	696548,60	606192,00	0,87	423038,00	118038,00
78299,00	78299,00	78946,15	74272,00	0,95	54299,00	503,00

217623,00	217623,00	218631,16	148577,00	0,68	113225,00	5752,00
119984,00	119984,00	120928,67	90968,00	0,76	19479,00	25134,00
338665,00	338665,00	336791,76	205302,00	0,61	55001,00	128894,00
122143,00	122143,00	119731,84	85695,00	0,70	34818,00	31088,00
559136,00	559136,00	637935,05	524965,00	0,94	451030,00	15496,00
284330,00	284330,00	300224,31	263203,00	0,93	194337,00	3584,00
43411,00	43411,00	44398,00	29986,00	0,69	12750,00	2921,00
841247,00	841247,00	844654,68	685562,00	0,81	372724,00	145241,00
60696,00	60696,00	63914,65	37346,00	0,62	13647,00	14618,00
1438504,00	1438504,00	1461961,64	1272260,00	0,88	964753,00	492879,00
351358,00	351358,00	352712,88	281067,00	0,80	82474,00	36464,00
102952,06	102952,06	101122,17	92705,06	0,90	48341,06	6600,00
84641,00	84641,00	81953,19	72060,00	0,85	41242,00	3255,00
271745,00	271745,00	280213,55	222212,00	0,82	101680,00	69797,00
442560,00	442560,00	487760,72	371482,00	0,84	153801,00	151858,00
183447,00	183447,00	180862,70	176421,00	0,96	100007,00	4869,00
863626,00	863626,00	907758,07	752110,00	0,87	599128,00	108912,00

liabili-ind	liabilind,equalis	assets-valueland	asets-valuela	assets-valuelandind,e	indoutput	indinput
176397,05	166158,75	501813,00	399862,38	392784,64	1,26	1,25
148241,23	155226,51	577392,00	389072,85	398689,41	1,19	1,19
190405,69	185612,18	398540,00	334976,71	332345,88	1,19	1,19
42,78	26,67	38861,00	33925,72	34684,93	1,15	1,15
10150,77	9780,97	187773,00	158059,59	151239,76	1,19	1,19
72758,73	67315,76	272621,00	149445,17	144714,45	1,83	1,82
159495,99	158668,96	385303,00	326019,14	318468,82	1,18	1,18
25853,59	25436,08	150222,00	125004,91	125044,01	1,55	1,55
22764,66	21920,95	235492,00	205579,48	201104,41	1,15	1,15
4775,48	4189,90	260899,00	213050,24	215440,62	1,22	1,22
16137,82	15083,72	95262,00	58694,30	59177,02	1,62	1,62
201189,50	195472,51	676636,00	464473,45	460397,86	1,46	1,46
36069,63	32013,74	132998,00	76285,11	69832,27	1,85	1,85
743535,45	705133,37	874071,00	739525,07	716217,82	1,18	1,18
66562,55	58040,27	399212,00	309271,07	308902,32	1,29	1,29
7359,56	7124,15	87226,00	62586,05	61965,74	1,32	1,32
3014,02	2733,95	53646,00	43197,96	42682,40	1,24	1,24
113230,03	110793,04	272877,00	216005,58	208553,17	1,26	1,26
206125,08	232892,97	499080,00	393353,32	426744,61	1,27	1,27
9057,51	7898,98	115066,00	91800,52	93134,39	1,26	1,26
163984,03	189540,61	430337,00	327118,97	375146,09	1,32	1,32
155920,44	160698,76	477024,00	385706,91	391340,11	1,24	1,24
162211,78	156074,17	584550,00	408305,97	401231,59	1,18	1,18
180818,66	184411,61	387811,00	329715,05	329478,17	1,18	1,18
10,57	29,90	40232,00	35444,13	34980,60	1,14	1,14
9411,18	9631,75	169630,00	144419,93	148408,83	1,17	1,17
61872,79	65326,92	247120,00	139983,73	141216,90	1,77	1,77
157841,92	159665,67	363418,00	310918,49	318492,93	1,17	1,17
25018,57	25532,67	154088,00	125083,11	125890,38	1,51	1,51
21077,25	21570,51	223196,00	196629,34	201247,51	1,14	1,14
3604,33	4039,36	263259,00	217830,99	208254,38	1,21	1,21
14029,61	15215,46	94174,00	59659,75	59774,26	1,58	1,58
189755,52	195858,47	649873,00	456322,27	460432,23	1,42	1,42
27957,84	30903,19	108404,00	63379,44	67988,69	1,79	1,79
666731,29	700765,03	809971,00	692910,56	700524,07	1,17	1,17
49518,00	53111,70	391851,00	308533,57	298348,48	1,27	1,27
6888,75	7050,38	84795,00	61345,43	61378,92	1,30	1,30
2453,89	2578,38	51637,00	42166,83	42576,38	1,22	1,22
108356,05	112214,05	250266,00	201100,76	208054,20	1,24	1,24
259660,85	243246,00	544389,00	460135,90	435265,55	1,25	1,25
6740,45	6970,35	116704,00	94468,26	89835,74	1,24	1,24
215097,19	196648,68	421957,00	423173,20	387707,72	1,29	1,29
149778,78	158559,84	472302,00	388451,03	371565,86	1,22	1,22
157769,49	156815,72	552439,00	406315,94	406481,36	1,16	1,16
182010,47	180366,71	376838,00	323742,75	325305,03	1,16	1,16
36,34	30,67	40136,00	35571,95	35869,00	1,13	1,13
9333,30	9215,41	165378,00	142746,97	139153,57	1,16	1,16
61349,25	60928,69	228624,00	134221,81	137321,33	1,71	1,70

161659,10	160194,47	370616,00	318541,15	316254,81	1,16	1,16
25725,87	25648,92	150481,00	127583,10	122443,71	1,46	1,46
20869,61	20647,33	226201,00	201533,72	198818,09	1,12	1,12
3738,29	3854,68	232713,00	193881,90	201031,51	1,20	1,20
15478,96	13910,67	92314,00	60968,75	59043,38	1,51	1,51
196630,40	198667,92	644768,00	460500,99	470548,22	1,40	1,40
28682,09	28692,62	106796,00	64301,53	64709,63	1,74	1,74
692028,36	690188,34	772443,00	669136,57	701201,92	1,15	1,15
43254,55	45576,05	348165,00	277240,80	288141,10	1,26	1,26
6902,82	6953,15	79831,00	60205,29	60314,27	1,28	1,28
2267,22	2508,76	51105,00	42364,34	42483,61	1,21	1,21
115056,07	110958,36	255768,00	207056,25	207394,85	1,24	1,24
263952,07	264696,07	533088,00	452307,45	458554,64	1,22	1,22
5113,08	5943,17	101226,00	83238,44	87896,54	1,22	1,22
210864,81	208016,26	423564,00	412830,97	408073,87	1,27	1,27
169980,30	161052,95	406964,00	340539,65	353547,04	1,20	1,20
150465,90	150212,67	543564,00	404822,18	401571,82	1,15	1,15
178271,00	176916,63	370964,00	322457,31	325261,39	1,15	1,15
45,09	60,62	41389,00	36590,92	36297,12	1,13	1,13
8901,76	8450,24	150467,00	130293,81	123660,75	1,15	1,15
59564,04	60580,15	230702,00	137758,44	138018,88	1,67	1,67
161082,38	159184,74	369550,00	319304,79	317735,40	1,16	1,16
26202,33	26038,18	134623,00	114664,90	118929,62	1,44	1,44
19995,14	20906,17	223231,00	198291,20	204757,41	1,13	1,13
4221,43	4087,59	227132,00	191381,64	191619,10	1,19	1,19
12223,45	13475,81	84197,00	56501,65	58937,04	1,49	1,49
209617,85	209126,46	687526,00	494821,40	486309,04	1,39	1,39
29437,94	28305,12	109430,00	66447,90	64379,97	1,72	1,72
711805,36	706887,13	852238,00	741558,61	720395,98	1,15	1,15
43955,62	43634,34	344219,00	278648,93	278809,65	1,24	1,24
7067,90	7000,99	81888,00	59392,11	59928,68	1,27	1,27
2805,16	2610,63	50904,00	42919,67	43289,01	1,19	1,19
109462,95	109695,92	263867,00	214027,55	210824,67	1,23	1,23
270475,31	266909,26	544357,00	463220,58	453856,63	1,20	1,20
5975,99	5445,65	103781,00	85982,91	86194,79	1,21	1,21
198086,79	198517,91	417731,00	388217,44	394086,84	1,24	1,24
163399,76	165896,15	391135,00	331650,46	338568,59	1,18	1,18
142402,62	143633,81	518606,00	393577,35	445678,22	1,14	1,14
170468,41	172721,20	372704,00	329584,11	339255,71	1,13	1,13
100,44	150,85	41689,00	36728,50	36176,69	1,14	1,14
7115,65	7312,50	112495,00	97941,46	107458,29	1,15	1,15
60827,17	59525,44	235300,00	142076,39	141754,05	1,66	1,66
154812,73	156854,54	360903,00	315360,27	322045,70	1,14	1,14
26186,36	26187,38	131760,00	114540,86	115702,96	1,41	1,41
21853,75	21366,11	223978,00	214447,31	195055,61	1,04	1,04
4303,05	3764,89	222945,00	189593,77	180354,53	1,18	1,18
12725,03	12246,48	88351,00	59340,72	58858,57	1,49	1,49
221131,13	215078,75	698434,00	503604,73	510108,24	1,39	1,39
26795,33	28418,13	102748,00	62390,47	66451,15	1,72	1,72
716827,66	717227,41	855825,00	750492,74	747116,43	1,14	1,14

43692,86	43716,44	338776,00	280539,23	287934,71	1,21	1,21
7032,27	7139,74	82354,00	60188,65	60345,08	1,26	1,26
2759,50	2731,00	51829,00	44583,02	46990,49	1,16	1,16
104568,74	104337,27	256364,00	211390,21	208937,87	1,21	1,21
266300,42	266596,31	527429,00	446041,84	448041,42	1,18	1,18
5247,89	4792,66	106785,00	89363,02	85005,33	1,20	1,20
186602,12	185247,11	407829,00	381212,11	394507,28	1,23	1,24
164308,38	163363,60	401159,00	343515,66	335310,88	1,17	1,17
138032,91	139006,58	732533,00	538635,13	479317,09	1,11	1,11
169424,19	166524,77	406012,00	365725,73	349106,50	1,11	1,11
307,03	247,86	40712,00	35210,66	35583,68	1,16	1,16
5920,10	6673,18	108370,00	94139,60	95858,90	1,15	1,15
58185,10	55701,59	233958,00	145427,33	140562,30	1,61	1,61
154668,50	154168,87	377190,00	331472,05	327326,89	1,14	1,14
26173,46	25949,11	136109,00	117903,13	114923,00	1,36	1,36
22249,43	22166,60	180291,00	172428,32	186360,93	1,05	1,05
2770,19	3238,74	186545,00	160088,20	167372,47	1,17	1,17
11790,96	11904,97	89604,00	60733,35	60505,41	1,48	1,48
214487,26	212500,08	718014,00	531898,60	519486,26	1,35	1,35
29021,11	27856,67	114093,00	70515,07	67111,95	1,69	1,69
723049,22	735215,00	852300,00	749297,94	757652,07	1,14	1,14
43500,85	43171,83	360014,00	304615,96	296582,40	1,18	1,18
7319,07	7203,35	83268,00	61454,48	59533,73	1,26	1,26
2628,35	2905,91	61721,00	53468,79	48088,71	1,15	1,15
98980,10	100798,30	240340,00	201395,85	205962,30	1,19	1,19
263013,19	260414,68	531477,00	434861,82	440307,69	1,16	1,16
3154,08	4147,22	94773,00	79670,05	83878,96	1,19	1,19
171052,42	173052,52	402355,00	414092,31	401394,06	1,21	1,21
162382,67	163531,94	381408,00	330766,53	332798,77	1,15	1,15
136584,21	141023,36	702388,00	505738,79	538843,72	1,09	1,09
159681,72	159155,07	383212,00	352009,66	347258,15	1,09	1,09
336,12	372,00	41221,00	34811,88	34798,91	1,18	1,18
6983,79	6528,40	109487,00	95495,65	104457,19	1,15	1,15
48092,50	49913,44	207489,00	134183,17	133645,14	1,55	1,55
153025,38	152210,15	378438,00	335148,36	329231,41	1,13	1,13
25487,52	27246,80	129237,00	112325,01	114399,55	1,32	1,32
22396,60	20946,35	177877,00	172207,15	165079,60	1,03	1,03
2642,97	2647,89	175623,00	152435,43	150757,06	1,15	1,15
11198,91	11150,57	89440,00	61442,16	62091,98	1,46	1,46
201881,84	196566,43	694166,00	522955,44	513131,99	1,33	1,33
27753,56	27401,91	108932,00	68430,30	67044,84	1,66	1,66
765768,11	738070,51	868337,00	773165,53	751366,26	1,12	1,12
42321,78	42194,57	354246,00	304592,00	300264,20	1,16	1,16
7258,70	7099,32	77323,00	56958,06	58291,67	1,25	1,25
3329,88	3159,30	52170,00	46214,31	48238,22	1,13	1,13
98846,06	99521,20	238661,00	205100,84	206230,82	1,16	1,16
251930,43	246800,21	529376,00	440019,40	425502,86	1,15	1,15
4039,70	3356,19	96719,00	82603,82	81892,86	1,17	1,17
161503,02	167832,26	406065,00	408877,76	413982,82	1,19	1,19
163904,79	162323,29	366525,00	324114,13	323823,72	1,13	1,13

148452,96	139225,88	799054,00	572157,25	532115,20	1,08	1,08
148359,29	151429,09	347553,00	324039,08	331485,74	1,07	1,07
472,86	429,67	40854,00	34374,18	31786,76	1,19	1,19
6681,31	6838,06	142028,00	123736,31	116728,33	1,15	1,15
43462,70	44485,16	180357,00	121324,91	122247,61	1,49	1,49
148936,56	149773,84	358374,00	321073,82	322506,99	1,12	1,12
30079,40	27447,82	130467,00	112970,51	107666,33	1,28	1,28
18193,01	20343,25	152085,00	150603,33	157616,64	1,01	1,01
2530,53	2521,59	158550,00	139747,55	135792,18	1,13	1,13
10461,83	10740,59	90791,00	64100,43	64215,27	1,42	1,42
173330,19	181535,49	627195,00	484541,93	502186,43	1,29	1,29
25431,05	25391,96	94192,00	62189,15	62763,47	1,60	1,61
725394,19	736176,21	809932,00	731635,29	739107,48	1,11	1,11
40761,09	39742,78	332266,00	291584,64	291399,18	1,14	1,14
6720,18	6893,99	75987,00	56462,47	55892,20	1,22	1,22
3519,69	3160,85	51036,00	45031,57	45072,96	1,13	1,13
100737,46	101069,37	239770,00	212195,77	209763,20	1,13	1,13
225457,02	229050,04	461377,00	401627,37	408951,19	1,14	1,14
2874,78	3416,67	97192,00	83404,72	84474,59	1,17	1,17
170941,33	161801,88	384563,00	418978,39	409666,28	1,17	1,17
160682,43	160579,61	351645,00	316590,50	319693,55	1,11	1,11
132640,47	136427,87	703817,00	518449,56	531636,00	1,08	1,08
146246,25	145416,46	337870,00	318408,48	318462,57	1,06	1,06
480,03	525,11	30862,00	26174,21	29063,51	1,18	1,18
6849,08	6818,71	150339,00	130953,02	126990,86	1,15	1,15
41900,28	45090,32	156901,00	111234,75	122317,80	1,41	1,41
147359,59	147302,36	344183,00	311298,78	311439,45	1,11	1,11
26776,52	28259,59	112016,00	97703,47	101120,33	1,25	1,25
20440,14	20173,85	149275,00	150039,43	147466,97	1,00	0,99
2391,28	2851,03	128620,00	115193,56	117413,22	1,12	1,12
10561,02	10694,43	90193,00	67103,22	64711,38	1,34	1,34
169394,46	163568,32	616506,00	499061,92	489008,40	1,24	1,24
22991,26	25003,75	82097,00	57670,97	60162,35	1,51	1,51
717366,34	709947,39	787273,00	712521,60	696803,89	1,10	1,10
36145,48	38479,04	311107,00	278020,89	290001,59	1,12	1,12
6703,08	6997,56	73034,00	54256,06	55354,97	1,19	1,19
2632,98	2869,80	49969,00	43972,98	43941,84	1,14	1,14
103624,59	100609,34	233407,00	211992,99	208221,97	1,10	1,10
209762,66	208585,87	413947,00	385206,81	380835,15	1,12	1,12
3335,51	3042,93	100820,00	87415,25	84882,37	1,16	1,16
152961,28	153240,48	364849,00	401142,67	398363,14	1,15	1,15
157151,62	150965,84	347082,00	318376,03	306184,65	1,09	1,09
128190,17	130982,81	665528,00	504301,21	512855,79	1,09	1,09
141643,84	139065,46	329937,00	312940,15	308256,94	1,05	1,05
622,44	518,41	31203,00	26642,14	26136,72	1,17	1,17
6925,74	6768,67	144759,00	126283,24	133271,60	1,15	1,15
49907,96	44930,26	186177,00	134393,74	119450,15	1,39	1,39
145610,92	143069,61	330330,00	301945,74	305728,94	1,09	1,09
27922,83	30681,31	101952,00	92687,01	99663,96	1,22	1,22
21888,39	21762,00	145622,00	141758,14	144245,82	1,03	1,03

3631,27	3357,49	108170,00	97298,56	102833,70	1,11	1,11
11060,45	10771,97	82711,00	62930,49	63033,34	1,31	1,31
147980,32	157162,33	576341,00	483421,34	487634,48	1,19	1,19
26588,96	25290,85	86655,00	60626,94	58775,61	1,51	1,51
687081,65	665794,43	707389,00	646254,78	650548,29	1,09	1,09
38530,55	37752,67	333249,00	300399,25	288146,48	1,11	1,11
7569,44	7419,29	67532,00	55346,37	55799,42	1,17	1,17
2456,73	2949,80	48351,00	42820,98	41830,17	1,13	1,13
97465,97	97644,26	220028,00	200477,16	207390,49	1,10	1,10
190537,92	187868,78	340361,00	355671,27	364198,84	1,11	1,11
2918,50	3046,80	97686,00	83827,15	96674,19	1,17	1,17
135818,83	139895,90	324899,00	374968,35	366393,00	1,13	1,13
135063,45	141634,23	307531,00	283587,42	292266,29	1,08	1,08
132117,77	130099,28	700651,00	515816,61	517646,92	1,06	1,06
129306,30	132085,51	303760,00	293422,20	298019,71	1,04	1,04
452,76	475,67	29225,00	25593,81	25852,73	1,14	1,14
6531,19	6616,89	163204,00	142578,53	137163,63	1,14	1,14
42982,54	43150,61	156422,00	112721,97	117566,82	1,39	1,39
136238,33	138045,40	332277,00	303942,30	302866,61	1,09	1,09
37344,57	33996,61	127671,00	108601,40	99924,69	1,18	1,18
22957,47	21248,37	151699,00	140939,89	135757,14	1,08	1,08
4049,92	3703,91	104972,00	96008,98	96168,11	1,09	1,09
10694,45	11129,28	80278,00	59066,30	60098,22	1,36	1,36
154112,22	149903,36	564862,00	480420,19	480869,29	1,18	1,18
26292,34	25151,51	93200,00	58028,93	57968,54	1,68	1,68
592935,31	618259,59	647519,00	592868,47	602386,30	1,09	1,09
38582,00	37448,23	311395,00	286019,30	288491,93	1,09	1,09
7985,36	7670,35	83603,00	57795,85	56049,60	1,12	1,12
3759,70	3180,43	43218,00	38696,56	41636,43	1,12	1,12
91842,22	92756,76	226129,00	209701,32	207370,19	1,08	1,08
163305,77	174933,55	363067,00	351718,44	345894,08	1,09	1,09
2886,39	3029,08	133867,00	118780,17	93413,61	1,13	1,13
130907,58	131069,97	297383,00	323067,96	343774,67	1,11	1,11
132687,60	130057,77	292463,00	274835,43	269581,56	1,06	1,06
129989,89	133509,61	637728,00	532822,94	543006,53	1,04	1,04
125306,39	125878,19	295160,00	287696,78	288213,66	1,03	1,03
351,81	392,00	27711,00	25322,24	25775,94	1,09	1,09
6393,73	6354,41	159656,00	142629,13	140446,61	1,12	1,12
36561,34	37206,00	137053,00	105584,74	102999,22	1,30	1,30
132286,94	132179,56	323286,00	302711,79	297465,69	1,07	1,07
36722,42	33722,07	110663,00	98485,65	95742,08	1,12	1,12
18899,25	19788,07	134505,00	124573,37	125696,63	1,08	1,08
3430,56	3697,05	101647,00	95196,80	97476,44	1,07	1,07
11632,93	9206,02	72225,00	58297,85	51522,08	1,24	1,24
147617,53	147381,64	541892,00	478766,33	469500,65	1,13	1,13
22573,23	23159,50	79419,00	55249,74	55319,19	1,50	1,50
574761,81	559829,58	606448,00	568035,65	555158,40	1,07	1,07
35232,16	35479,41	297994,00	279057,25	281038,60	1,07	1,07
7456,27	7257,66	71146,00	55006,58	54721,49	1,08	1,08
3324,87	3381,06	47635,00	43391,77	41287,74	1,10	1,10

88962,10	87110,73	221804,00	211932,09	202257,54	1,05	1,05
170956,95	165571,25	343299,00	330292,53	347316,35	1,05	1,05
3282,35	3056,06	83751,00	77633,51	90412,42	1,08	1,08
126483,51	124111,94	350833,00	333287,68	317097,07	1,08	1,08
122422,26	124039,47	261322,00	250321,82	256289,18	1,04	1,04
138421,18	135448,37	659184,00	580380,04	557169,08	1,01	1,01
123021,88	121572,73	285820,00	283521,99	281741,94	1,01	1,01
371,44	360,74	27945,00	26411,77	25540,84	1,06	1,06
6138,31	6084,82	147347,00	136132,17	128237,67	1,08	1,08
32074,14	33430,69	104718,00	90690,96	98680,96	1,15	1,15
128013,40	129896,42	297559,00	285742,99	290089,42	1,04	1,04
27099,22	32311,72	81215,00	80139,21	89703,73	1,06	1,06
17507,49	17133,75	119030,00	111576,63	114474,82	1,07	1,07
3610,67	3500,84	105466,00	101223,55	99961,23	1,04	1,04
5290,67	6831,40	42450,00	37202,08	42999,41	1,14	1,14
140415,16	143828,16	501067,00	449315,42	466562,62	1,12	1,12
20612,93	20742,72	63037,00	52678,88	53658,71	1,25	1,25
511791,62	528001,80	527734,00	504571,09	515416,73	1,05	1,05
32624,07	34224,11	290456,00	278039,25	277385,66	1,04	1,04
6331,35	6677,82	62246,00	51362,05	52689,78	1,04	1,04
3058,62	2949,45	44539,00	41774,88	42103,17	1,07	1,07
80527,87	82438,64	188572,00	185139,20	191706,99	1,02	1,02
162451,03	161393,30	363585,00	359938,07	329563,44	1,02	1,02
2999,43	3297,87	77482,00	74823,59	77739,74	1,04	1,04
114944,72	121410,43	311417,00	294935,57	306263,51	1,05	1,05
117008,57	119900,94	248785,00	243710,29	246251,37	1,02	1,02
137934,04	137993,07	598076,00	558304,25	557882,77	1,00	1,00
116389,91	119149,93	275142,00	274007,06	277521,35	1,00	1,00
358,97	411,14	25445,00	24888,50	25100,09	1,02	1,02
5722,42	5870,91	110295,00	105951,72	115493,96	1,04	1,04
31656,59	29621,58	105665,00	99767,17	96987,71	1,06	1,06
129388,92	128765,44	287274,00	281813,49	283740,16	1,02	1,02
33113,52	30433,58	94245,00	90486,34	85983,51	1,03	1,03
14994,51	15999,33	110619,00	107274,46	108985,70	1,03	1,03
3461,29	3551,99	105547,00	103463,33	98226,63	1,02	1,02
3570,60	3927,42	35810,00	33498,30	33787,13	1,07	1,07
143451,80	143035,99	491388,00	471606,11	463148,18	1,04	1,04
19042,00	18090,98	56449,00	53047,50	50925,13	1,11	1,11
497451,97	500707,53	483023,00	473643,45	483988,51	1,02	1,02
34816,10	34634,72	282012,00	275060,49	273994,58	1,03	1,03
6245,86	6392,40	59756,00	51700,70	52557,91	1,03	1,03
2464,86	2926,16	42514,00	41142,86	42105,58	1,03	1,03
77825,95	76050,27	179697,00	178049,68	177751,29	1,01	1,01
150771,91	155026,98	295352,00	298459,70	315718,92	1,01	1,01
3611,82	3826,75	81817,00	80762,12	79675,24	1,02	1,02
122803,07	115553,26	296958,00	290567,27	283333,61	1,03	1,03
120272,00	118640,28	244722,00	244722,00	244216,15	1,00	1,00
137624,00	137779,02	534964,00	534964,00	546634,13	1,00	1,00
118038,00	117213,96	275035,00	275035,00	274521,03	1,00	1,00
503,00	430,99	24000,00	24000,00	24444,25	1,00	1,00

5752,00	5737,21	104398,00	104398,00	105174,86	1,00	1,00
25134,00	28395,29	100505,00	100505,00	100136,08	1,00	1,00
128894,00	129141,46	283664,00	283664,00	282738,75	1,00	1,00
31088,00	32100,76	87325,00	87325,00	88905,67	1,00	1,00
15496,00	15245,26	108106,00	108106,00	107690,23	1,00	1,00
3584,00	3522,65	89993,00	89993,00	96728,17	1,00	1,00
2921,00	3245,80	30661,00	30661,00	32079,65	1,00	1,00
145241,00	144346,40	468523,00	468523,00	470064,55	1,00	1,00
14618,00	16830,00	47049,00	47049,00	50048,25	1,00	1,00
492879,00	495165,49	473751,00	473751,00	473697,23	1,00	1,00
36464,00	35640,05	268884,00	268884,00	271972,25	1,00	1,00
6600,00	6422,93	54611,00	54611,00	53155,85	1,00	1,00
3255,00	2859,93	43399,00	43399,00	42270,93	1,00	1,00
69797,00	73811,48	170065,00	170065,00	174057,34	1,00	1,00
151858,00	151314,95	288759,00	288759,00	293609,35	1,00	1,00
4869,00	4240,41	83440,00	83440,00	82101,06	1,00	1,00
108912,00	115857,54	264498,00	264498,00	277532,63	1,00	1,00

exchangerat	income1employun	econsustain(benchmark)	econsustainable
1,00	1023,39	405,01	1,00
0,80	152,13		1,00
1,00	404,26		1,00
1,00	988,70		1,00
1,00	635,83		1,00
1,00	58,04		1,00
1,00	364,01		1,00
1,29	405,01		1,00
1,00	502,48		1,00
1,00	1319,53		1,00
1,00	147,35		1,00
1,00	457,27		1,00
1,06	112,19		1,00
1,00	1862,97		1,00
1,00	740,61		1,00
0,95	340,66		1,00
1,00	639,39		1,00
1,00	237,79		1,00
1,00	140,33		1,00
1,00	625,55		1,00
1,00	249,87		1,00
1,00	1018,68	402,86	1,00
0,82	139,03		1,00
1,00	402,86		1,00
1,00	940,47		1,00
1,00	608,64		1,00
1,00	39,24		1,00
1,00	331,41		1,00
1,23	382,94		1,00
1,00	485,86		1,00
1,00	1377,03		1,00
1,00	149,88		1,00
1,00	421,78		1,00
1,04	124,30		1,00
1,00	1943,99		1,00
1,00	697,71		1,00
0,94	337,86		1,00
1,00	578,22		1,00
1,00	232,22		1,00
1,06	145,95		1,00
1,00	577,84		1,00
1,30	233,03		1,00
1,00	908,17	378,95	1,00
0,86	140,47		1,00
1,00	393,98		1,00
1,00	935,09		1,00
1,00	601,76		1,00
1,00	37,87		1,00

1,00	319,26		1,00
1,24	350,42		1,00
1,00	503,28		1,00
1,00	1309,12		1,00
1,00	167,53		1,00
1,00	424,64		1,00
1,04	134,91		1,00
1,00	1797,86		1,00
1,00	700,32		1,00
0,96	327,53		1,00
1,00	565,28		1,00
1,00	217,39		1,00
1,04	168,46		1,00
1,00	436,97		1,00
1,24	213,88		1,00
1,00	844,89	379,72	1,00
0,86	163,23		1,00
1,00	363,91		1,00
1,00	959,94		1,00
1,00	521,34		1,00
1,00	18,19		1,00
1,00	314,12		1,00
1,23	340,60		1,00
1,00	478,05		1,00
1,00	1343,30		1,00
1,00	158,62		1,00
1,00	406,40		1,00
1,04	117,11		1,00
1,00	1524,02		1,00
1,00	625,77		1,00
0,92	322,32		1,00
1,00	524,34		1,00
1,00	221,38		1,00
1,03	126,79		1,00
1,00	395,52		1,00
1,15	192,43		1,00
1,00	866,53	401,20	1,00
0,86	178,02		1,00
1,00	405,93		1,00
1,00	939,58		1,00
1,00	522,16		1,00
1,00	48,34		1,00
1,00	336,02		1,00
1,23	332,91		1,00
1,00	467,38		1,00
1,00	1214,72		1,00
1,00	167,25		1,00
1,00	441,98		1,00
1,04	103,64		1,00
1,00	1509,29		1,00

1,00	655,12		1,00
0,92	349,73		1,00
1,00	468,17		1,00
1,00	254,38		1,00
1,00	115,78		1,00
1,00	401,20		1,00
1,15	213,77		1,00
1,00	976,86	390,23	1,00
0,82	182,56		1,00
1,00	473,12		1,00
1,00	973,81		1,00
1,00	453,32		1,00
1,00	78,01		1,00
1,00	384,95		1,00
1,18	350,28		1,00
1,00	454,85		1,00
1,00	1352,85		1,00
1,00	181,67		1,00
1,00	403,27		1,00
1,04	99,11		1,00
1,00	1571,93		1,00
1,00	655,16		1,00
0,93	383,69		1,00
1,00	455,13		1,00
1,00	283,81		1,00
0,95	114,42		1,00
1,00	386,47		1,00
1,25	260,39		1,00
1,00	1005,25	436,77	1,00
0,79	174,69		1,00
1,00	482,40		1,00
1,00	1004,50		1,00
1,00	475,62		1,00
1,00	107,82		1,00
1,00	433,48		1,00
1,15	353,81		1,00
1,00	445,12		1,00
1,00	1220,87		1,00
1,00	221,83		1,00
1,00	413,82		1,00
1,04	107,82		1,00
1,00	1422,00		1,00
1,00	759,92		1,00
0,92	417,41		1,00
1,00	440,07		1,00
1,00	304,39		1,00
0,95	135,45		1,00
1,00	441,10		1,00
1,20	342,19		1,00
1,00	1108,73	454,04	1,00

0,77	125,85		1,00
1,00	454,04		1,00
1,00	1162,74		1,00
1,00	521,70		1,00
1,00	121,60		1,00
1,00	474,30		1,00
1,11	297,87		1,00
1,00	480,22		1,00
1,00	1295,42		1,00
1,00	239,77		1,00
1,00	387,08		1,00
1,06	112,77		1,00
1,00	1383,81		1,00
1,00	777,35		1,00
0,91	420,73		1,00
1,00	454,59		1,00
1,00	365,35		1,00
0,99	146,91		1,00
1,00	489,81		1,00
1,28	373,21		1,00
1,00	997,11	374,95	1,00
0,79	92,98		1,00
1,00	358,50		1,00
1,00	1308,84		1,00
1,00	519,11		1,00
1,00	99,23		1,00
1,00	376,83		1,00
1,09	236,57		1,00
1,00	429,13		1,00
1,00	1284,20		1,00
1,00	226,29		1,00
1,00	349,60		1,00
1,06	100,38		1,00
1,00	1027,05		1,00
1,00	724,24		1,00
0,88	374,95		1,00
1,00	437,47		1,00
1,00	332,33		1,00
1,05	121,55		1,00
1,00	531,82		1,00
1,26	363,17		1,00
1,00	979,83	340,58	1,00
0,83	69,22		1,00
1,00	323,42		1,00
1,00	1416,34		1,00
1,00	543,32		1,00
1,00	83,02		1,00
1,00	340,58		1,00
1,11	212,96		1,00
1,00	384,06		1,00

1,00	1226,32		1,00
1,00	234,88		1,00
1,00	329,43		1,00
1,06	95,13		1,00
1,00	962,00		1,00
1,00	717,19		1,00
0,96	332,33		1,00
1,00	407,42		1,00
1,00	341,17		1,00
1,16	161,69		1,00
1,00	525,58		1,00
1,30	321,83		1,00
1,00	1016,77	384,24	1,00
0,78	89,05		1,00
1,00	389,37		1,00
1,00	1618,10		1,00
1,00	584,63		1,00
1,00	112,06		1,00
1,00	350,94		1,00
1,00	189,22		1,00
1,00	384,24		1,00
1,00	1436,77		1,00
1,00	262,79		1,00
1,00	445,23		1,00
1,04	115,89		1,00
1,00	877,42		1,00
1,00	810,04		1,00
0,78	317,07		1,00
1,00	386,79		1,00
1,00	365,64		1,00
1,05	201,80		1,00
1,00	547,56		1,00
1,21	317,36		1,00
1,00	1131,00	396,18	1,00
0,87	108,29		1,00
1,00	430,18		1,00
1,00	1704,72		1,00
1,00	646,42		1,00
1,00	122,54		1,00
1,00	411,76		1,00
1,00	201,15		1,00
1,00	393,09		1,00
1,00	1440,48		1,00
1,00	238,03		1,00
1,00	502,24		1,00
1,04	139,86		1,00
1,00	1121,17		1,00
1,00	861,90		1,00
0,84	355,13		1,00
1,00	382,53		1,00

1,00	396,18		1,00
1,01	211,59		1,00
1,00	453,34		1,00
1,03	279,81		1,00
1,00	1226,04	409,76	1,00
0,89	99,61		1,00
1,00	450,46		1,00
1,00	1720,59		1,00
1,00	639,60		1,00
1,00	145,56		1,00
1,00	416,82		1,00
1,05	176,35		1,00
1,00	406,75		1,00
1,00	1328,97		1,00
1,00	308,71		1,00
1,00	513,54		1,00
1,04	175,95		1,00
1,00	1330,05		1,00
1,00	813,24		1,00
0,86	363,31		1,00
1,00	364,97		1,00
1,00	409,76		1,00
1,01	165,83		1,00
1,00	481,63		1,00
1,00	254,54		1,00
1,00	1170,62	393,90	1,00
0,93	89,57		1,00
1,00	393,90		1,00
1,00	1640,41		1,00
1,00	671,27		1,00
1,00	127,98		1,00
1,00	376,66		1,00
0,99	131,02		1,00
1,00	417,51		1,00
1,00	1299,79		1,00
1,00	228,20		1,00
1,00	500,82		1,00
1,04	173,02		1,00
1,00	1236,77		1,00
1,00	738,21		1,00
0,89	335,76		1,00
1,00	361,69		1,00
1,00	400,44		1,00
1,02	97,74		1,00
1,00	445,79		1,00
1,00	213,56		1,00
1,00	1136,97	397,17	1,00
1,00	90,05		1,00
1,00	397,17		1,00
1,00	1741,13		1,00

1,00	666,27		1,00
1,00	140,52		1,00
1,00	367,21		1,00
1,00	123,19		1,00
1,00	418,35		1,00
1,00	1327,22		1,00
1,00	218,40		1,00
1,00	536,68		1,00
1,00	159,21		1,00
1,00	1128,21		1,00
1,00	754,05		1,00
1,00	354,67		1,00
1,00	338,83		1,00
1,00	432,33		1,00
1,00	97,99		1,00
1,00	506,63		1,00
1,00	208,71		1,00
